# Wind disasters adaptation in cities in a changing climate: A systematic review

**DOI:** 10.1371/journal.pone.0248503

**Published:** 2021-03-17

**Authors:** Yue He, Boqun Wu, Pan He, Weiyi Gu, Beibei Liu

**Affiliations:** 1 State Key Laboratory of Pollution Control & Resource Reuse School of Environment, Nanjing University, Nanjing, China; 2 Department of Environmental Systems Science, Swiss Federal Institute of Technology (ETH), Zürich, Switzerland; 3 Department of Earth System Science/Institute for Global Change Studies, Tsinghua University, Beijing, China; 4 School of Earth and Ocean Sciences, Cardiff University, Cardiff, United Kingdom; 5 The John Hopkins University-Nanjing University Center for Chinese and American Studies, Nanjing, China; Institute of Oceanology Chinese Academy of Sciences, CHINA

## Abstract

Wind-related disasters will bring more devastating consequences to cities in the future with a changing climate, but relevant studies have so far provided insufficient information to guide adaptation actions. This study aims to provide an in-depth elaboration of the contents discussed in open access literature regarding wind disaster adaptation in cities. We used the Latent Dirichlet Allocation (LDA) to refine topics and main contents based on 232 publications (1900 to 2019) extracted from Web of Science and Scopus. We conducted a full-text analysis to filter out focal cities along with their adaptation measures. The results show that wind disaster adaptation research in cities has formed a systematic framework in four aspects: 1) vulnerability and resilience of cities, 2) damage evaluation, 3) response and recovery, and 4) health impacts of wind disaster. Climate change is the background for many articles discussing vulnerability and adaptation in coastal areas. It is also embedded in damage evaluation since it has the potential to exacerbate disaster consequences. The literature is strongly inclined towards more developed cities such as New York City and New Orleans, among which New York City associated with Hurricane Sandy ranks first (38/232). Studies on New York City cover all the aspects, including the health impacts of wind disasters which are significantly less studied now. Distinct differences do exist in the number of measures regarding the adaptation categories and their subcategories. We also find that hard adaptation measures (i.e., structural and physical measures) are far more popular than soft adaptation measures (i.e., social and institutional measures). Our findings suggest that policymakers should pay more attention to cities that have experienced major wind disasters other than New York. They should embrace the up-to-date climate change study to defend short-term disasters and take precautions against long-term changes. They should also develop hard-soft hybrid adaptation measures, with special attention on the soft side, and enhance the health impact study of wind-related disasters.

## Introduction

As one of the world’s principal natural disaster types, wind disasters contribute to huge economic losses and human casualties [1]. They are responsible for destroying buildings and structures [2], killing plants on their paths [3], and inducing respiratory diseases in humans [4]. The Emergency Events Database (EM-DAT) recorded over 2,900 wind-related disasters in the past 120 years [5]. Among the recorded disasters, tropical cyclones alone recorded a frequency of 2,299, second only to riverine floods among all disaster subtypes. Apart from the high frequency, wind disasters are noteworthy for their devastating power. Disasters in the United States cost the country 160 billion USD between 1980 and 2019. These include the most expensive hurricane Katrina ($170 billion) and the deadliest Hurricane Maria that killed almost 3,000 people [6]. Therefore, there is an urgent need for adaptation measures to mitigate these adverse impacts, since cities are becoming more vulnerable due to increased development towards risky zones [7] and reduced protection from ecosystems [8]. Stocker et al. define adaptation as “the process of adjustment to actual or expected climate and its effects to either lessen or avoid harm or exploit beneficial opportunities”(pp. 76) [9]. It is demonstrated that proper adaptation measures are cost-effective in decreasing storm damages in the long-term [10, 11]. Effective track monitoring, early warning, and timely evacuation are principal measures to alleviate negative consequences [12]. Well-established watching and warning systems for tropical cyclones are in place at the national level [13]. And also at the city level in some countries such as Shanghai, China [14]. However, the combination of global warming and weather variability is going to increase uncertainty over future wind disasters. Climate change is expected to increase the frequency and intensity of wind-related storms [15–18]. For example, sea-level rise will shorten the return period of extreme floods caused by hurricanes from 100 years to between 3 to 20 years in New York City (NYC) [19]. It is estimated that global damage from tropical cyclones with climate change will be doubled in 2100 compared with the baseline scenario, concentrating in North America, the Caribbean region, and East Asia [20]. The reliability of these projections remains in doubt. Because researchers argue that existing databases are insufficient for long-term predictions due to the development of observational technologies and capabilities [21]. Thus, it is understandable that conflicting conclusions stating a decreasing global mean frequency of tropical cyclones exist [22]. Cities should not only take wind disasters as natural disasters when planning adaptation measures. They should also realize their potential to go beyond natural variability in the context of climate change.

Wind-induced damages on buildings are characterized by the so-called “coherent phenomenon” and “damage chain” [23]. The “coherent phenomenon” is described in such a way that once the eaves are damaged by wind, the roof materials will be blown away thereafter. Debris flying in the downwind direction may crash into buildings on its track, then similar consequences as a coherent phenomenon will happen, which is the so-called “damage chain”. These flying debris are also responsible for human causalities by directly hitting them or by forming obstacles on the road [24]. Nevertheless, the destructiveness of wind-related disasters comes from more than the wind itself as wind disasters can trigger secondary hazards. For example, tropical cyclones are multi-hazard complexes, which may occur together with storm surges, floods, and lightning. The intensity of some secondary hazards such as storm surges is also affected by the wind [25]. All these factors add to the complexity of wind disasters and the difficulty for cities to adapt. In this paper, we aim to address the following questions in the field of wind adaptation in cities: What are the major topics, aspects, and contents discussed in literature? What are the focal cities that are most often studied? What kinds of wind adaptation measures are often suggested or adopted? Has climate change been incorporated into wind adaptation? According to the Carbon Disclosure Project (CDP), an increasing number of cities have reported their concerns about increased wind risks due to climate change between 2012 and 2017. 109 reports out of 263 (41% of total) labeled serious or extremely serious risks. In 2019, 87% of wind risks had corresponding adaptation measures but they were yet to be fully completed [26]. Concerns about increasing wind speeds were also included. Contrary to popular belief, wind speed in a city can be faster than in rural areas despite the rough landscape of urban areas [27]. Ground-level wind speed increases significantly in streets between buildings [28]. These winds are harmful to pedestrians in terms of re-suspended particulates and flying debris [29]. Therefore, raised concerns as well as inadequate adaptation drive the academia to propose more effective suggestions and address the problems.

Although case studies that discuss urban adaptation to specific wind-related storms are available [30], a systematic description of adaptation to wind-related disasters at the city level remains absent. Early studies that reviewed mitigation to natural hazards have failed to embrace the concept of “adaptation” [31], at the same time, existing literature reviews put more emphasis on general adaptation to climate change (including climatic hazards) instead of focusing on specific ones regarding wind disasters [32–34]. Compared with responding to long-term climate change, responding to extreme events is even more important since those events are more salient and intense [35]. Our comprehensive description of adaptation to wind-related disasters will be useful in terms of systematically gathering pieces of knowledge and exploring innovative solutions for cities faced with severe wind disasters in the case of finite adaptive capacity.

In this paper, we explore detailed contents in open-access literature that discusses adaptation to wind-related disasters in cities. Open access articles and journals are developing more rapidly than the average development speed of all types of articles and journals. It is becoming a popular platform for emerging subjects and distributing literature [36, 37]. More importantly, open access articles are more advantageous in attracting stable social attention over time than non-open-access articles [38]. Since adaptation calls for public participation [39] and attention to the most disadvantaged population [40], open access articles are expected to provide these people with desirable and useful knowledge in a low-cost and equal way. Thus, we separated open access articles to see if they have formed a systematic knowledge system.

This paper reviews the adaptation of cities to wind-related disasters in a structured way, with a special focus in the context of climate change. First, we developed a structured research query for systematic review. Then we extracted, categorized, and analyzed the embedded topics based on topic modeling. Next, experts read through the final literature database and filtered out cities and adaptation measures. Then we summarized the frequency of cities and adaptation measures. We presented links between cities and topics, as well as between cities and adaptation measures. Finally, we concluded the important discoveries, limitations, and future directions.

## Methods

### A structured search query for web of science and scopus

To answer the question of “what is studied” in a broad scientific field, we designed advanced search queries for Web of Science (WoS) and Scopus to establish a literature database. Bibliometric analyses use these two search engines frequently. WoS and Scopus have a strong bias towards Natural Sciences and Engineering in their contents [41]. They share some identical journals while at the same time, each also has unique ones [42]. On one hand, WoS contains more subject areas [43] and has the oldest literature that can be traced back to 1900 [44]. On the other hand, Scopus covers many more unique journals [42] and non-English publications [43]. We combined these two search engines to make up for each other’s deficiencies.

When referring to the research questions, keywords related to three aspects (“city”, “wind disasters”, and “adaptation”) were developed. We did not include “climate change” in the list of keywords for two reasons. The first reason is not to limit wind disasters to climatic ones as the wind disaster itself is first and foremost, a type of natural disaster. Whether climate change could alter the frequency and intensity of wind disasters is yet to be determined. Therefore, adaptation to wind disasters does not necessarily take climate change into account. The second reason is not to connect wind disasters and climate change intentionally. We expect to see climate change emerge as the new keyword in the results to examine if current research has integrated climate change into adaptation. Based on the authors’ experiences from browsing the literature, potentially relevant words were gradually gathered and refined. The results were sorted according to relevance after each search query was performed. Experts screened the titles and abstracts of the one hundred most relevant pieces of literature to examine the relevance and validity of the keywords. Phrases were connected by “OR” within the category and by “AND” between categories. The period was from the beginning of 1900 to the end of 2019. We chose all languages and all document types within the timeframe to comprehensively include all possibly relevant publications. It is important to note here that only literature with open access was downloaded together with their abstracts, authors, journals and year of publication, etc. The composition of keywords and the final search queries are provided in the S1 Appendix. After cleaning up the duplicate literature, the primary corpus was imported to ABSTRACKR, a website developed for screening publications for systematic reviews. Irrelevant publications were identified and deleted after screening through the abstracts of all literature. Before the formal screening, two experts reviewed 20 identical pieces of literature separately. Then, the results were compared and discussed to clarify the principles for literature to be reserved. Generally, literature that has nothing to do with cities, wind disasters, or fails to focus on adaptation-related themes, was removed from the corpus. Currently, there is no standard quality assessment for individual environmental studies. Tools applied in the medical field are not suitable for this review because we target descriptive contents of literature that are relevant to our topic. Our quality control is reflected in the process of literature screening where we made judgments according to three inclusion criteria for each article. They are i) The article’s discussion unfolds at the city level. ii) The main disaster discussed in the article are wind-related disasters. For example, tropical cyclones, tornadoes, sandstorms, and dust storms. iii) Adaptation should be the subject of the article.

### Topic modelling and content analysis

We ran a topic model based on the Latent Dirichlet Allocation (LDA) to excavate topics discussed by the literature corpus. LDA is one of the most popular techniques in data mining, and latent topic exploring is applied across multiple fields [45]. LDA realizes dimensionality reduction to preserve the essential latent information by assigning a fixed number of topics and corresponding possibilities to each literature [46]. Previous literature reviews have done topic extraction in a similar way [47, 48]. LDA is a suitable algorithm for the analysis because it does not allow subtracted elements to be negative [49], and it assumes Dirichlet prior at the document level, therefore making it more flexible in representing and interpreting the literature corpus [50].

As a pre-processing measure, we deleted letters that do not belong to the English language system (literature in Spanish has abstracts in both Spanish and English) and meaningless words such as stop words in detected root words. The model then calculated frequencies for the remaining root words and used them to generate topics with root words (set as 6 in this study) assigned to the same topic according to the frequency of two words occurring together. Each piece of literature was presented with the topics and corresponding possibilities. The topic with the highest possibility was regarded as the final topic of that literature. We ran the model repeatedly, varying the number of topics from three to thirty-two, and compared the meaningfulness of topics. Finally, the meaning of each topic was organized by authors subjectively.

Text analysis was carried out simultaneously with the establishment of the topic modeling. We referred to the fifth Intergovernmental Panel on Climate Change (IPCC) report [51] to group adaptation measures into three categories and ten subcategories. Information about cities and adaptation measures was identified via manual read-through of publications. We designed a template for filling in the targeted information (S2 Appendix).

**Fig 1:**
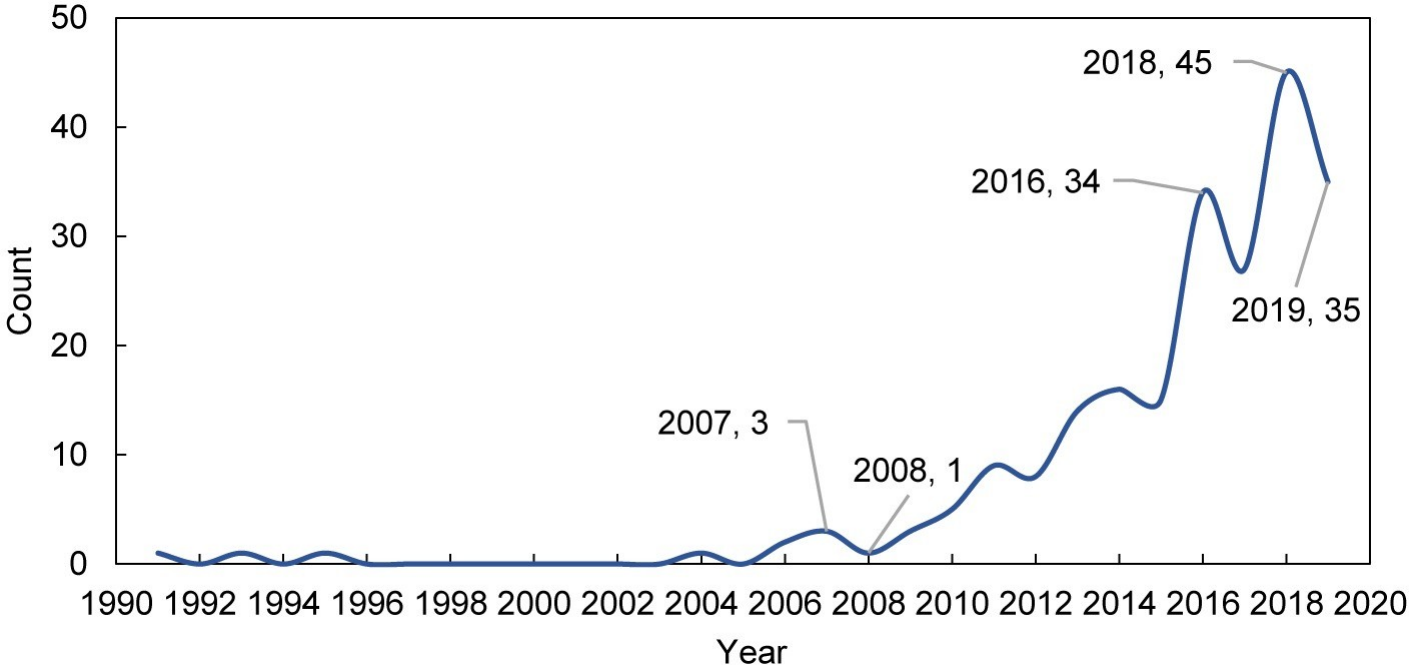
The number of annual publications between 1990 and 2019.

232 articles were selected (S1 Table). There was a total of 221 articles downloaded with abstracts. The earliest literature available can be traced back to 1991, followed by a more than ten-year development stagnation (Fig 1). It was not until 2007 that the annual number of publications about wind disaster adaptation in cities started to show an increasing trend, albeit at a slow pace. This is understandable since adaptation measures taken by developed countries were still very limited even in 2011 [32].

## Results and discussion

### Statistics of the literature corpus

**Fig 2:**
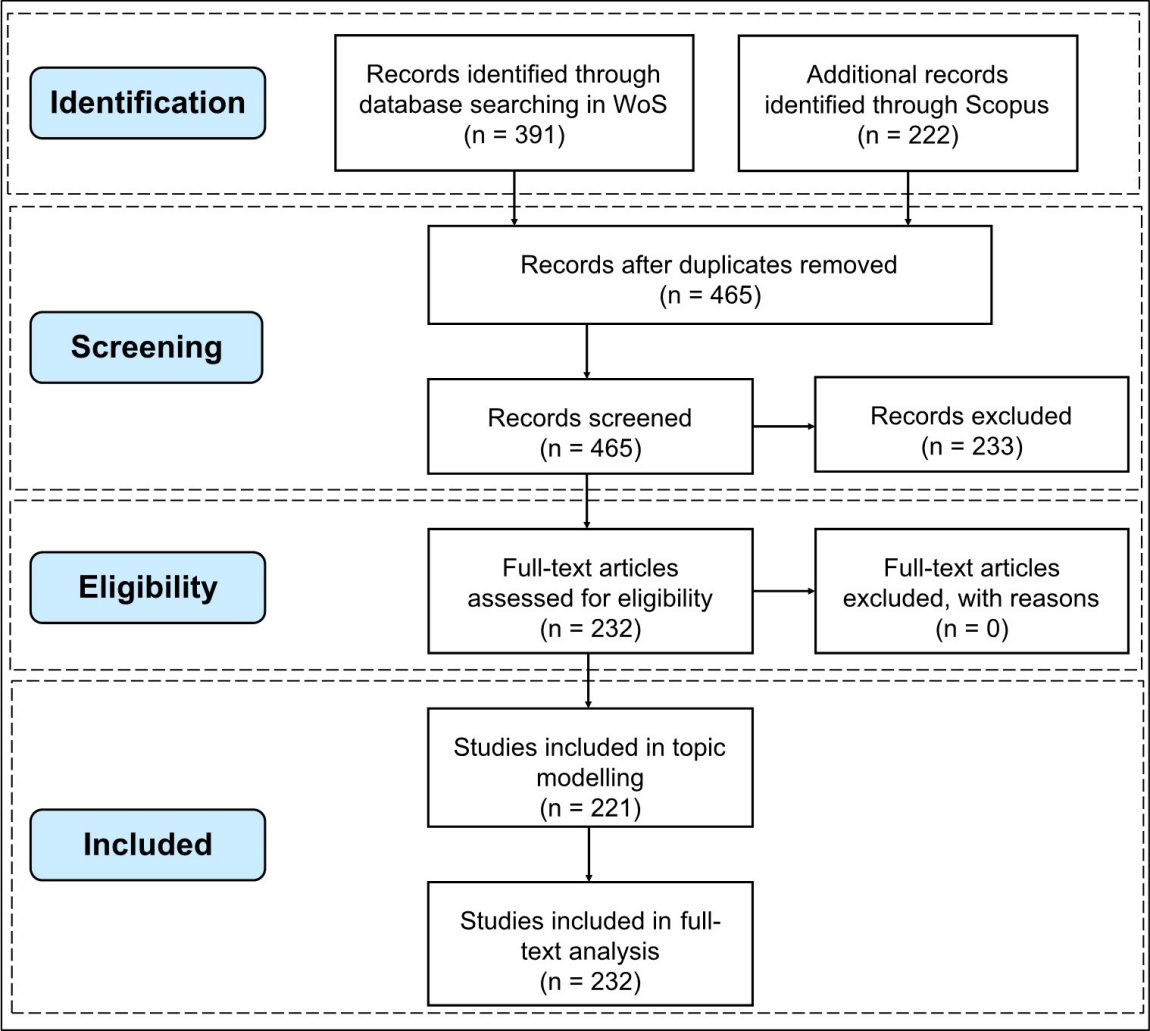
PRISMA flow diagram for literature search.

**Fig 3:**
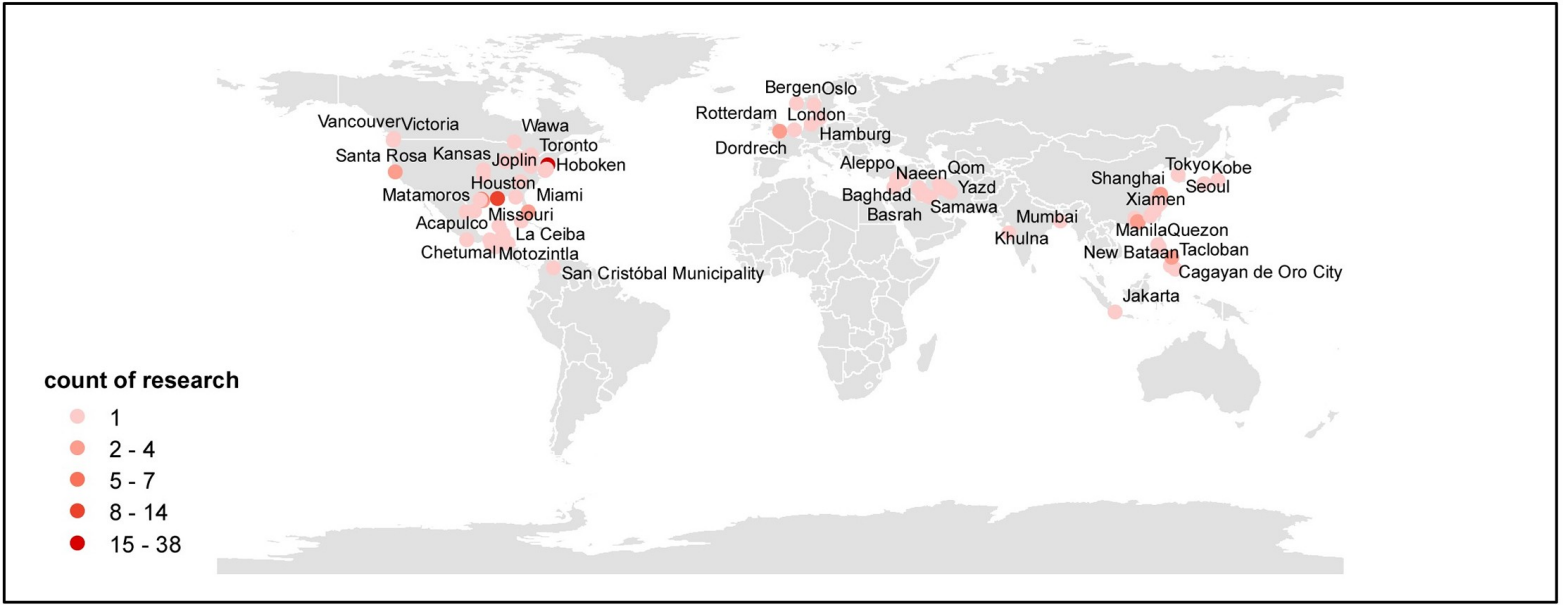
Geographical distribution of cities mentioned in publications and their frequencies.

Fig 2 shows the steps of establishing the corpus. 87% of the literature consists of academic articles (total of 202), accompanied by very few conference proceedings and reports. The inherent characteristics of WoS and Scopus determine such distribution of literature types [41]. Case studies make up most of the academic articles. We retrieved the names of all cities that were studied in the literature and the frequency that cities were mentioned and formed a map displaying the geographical distribution of these cities (Fig 3). We find that the cities under study are concentrated mostly in the continents of the Northern Hemisphere, such as East Asia, South Asia, and North America containing most cities. There is a prominent research bias towards NYC, which has been mentioned 38 times in total. The topography and coastal proximity of NYC make it more vulnerable to wind disasters, which may trigger other extreme events such as floods [52]. As a megacity, NYC is appropriate for city-level case studies. The well-developed social systems in NYC may play a role in providing relevant research with abundant data and sufficient funding. Most importantly, the sudden shock of Hurricane Sandy triggered a tipping point that raised awareness of building resilient cities against severe natural disasters and climate risks [30]. The other six most frequently mentioned cities are New Orleans (14), Houston (7), Hong Kong (4), Galveston (3), Shanghai (3), and Tacloban (3).

### Major aspects and contents in wind disaster adaptation

#### Statistics and categorization of topics

We selected nine topics for final analyses after generating and comparing multiple numbers of topics with LDA. Topics under this number cover more areas than smaller numbers, and at the same time, avoid duplicate themes. We subjectively summarized the contents of topics according to stemmed words from LDA (Table 1). The number of each topic distributes evenly. 34 publications are talking about the vulnerability and adaptation of coastal areas in the framework of future climate change, which is the topic of the highest frequency. Although we did not include climate-related keywords in the literature search, it still appears in one of the nine topics, which indicates the necessity of incorporating climate change into the studies of wind disaster adaptation. 21 publications are highlighting resilient measures in the local context, including those taken by communities. 20 publications talk about the damages of extreme events on cities, especially the damages caused by cyclones. 19 publications apply models and big data to analyze the impacts of disasters on the social networks and urban systems. 26 publications provide assessments of flood hazards and risks in urban areas. 24 publications relating to models focus on simulations of windstorms and water surges. 28 publications talk about cities’ project designs in the recovery process, mainly focusing on New Orleans. 23 publications discuss how humans respond to hurricanes and other disasters. 26 publications are related to the health impacts of wind disasters, mostly from Hurricane Sandy.

**Table 1:** Summary of stemmed roots, nine topics, four categories and the distribution of each topic in the literature corpus.

Reference number	Topics	Stemmed word roots	Aspects	Number of publications	Marginal percentage
1	Vulnerability and adaptation of coastal areas in the background of future climate change	Vulner, chang, climat, coastal, adapt, futur	Vulnerability and resilience of cities (topic 1 and 2)	34	24.9%
2	Development of social resilience of urban communities	Resili, urban, communiti, social, develop, local		21	
3	Damages of extreme cyclone events on cities and regions	Citi, event, damag, extrem, region, cyclon	Damage evaluation (topic 3, 4, 5, and 6)	20	40.3%
4	Use of models and timeline data to analyze networks and systems	System, model, data, time, network, event		19	
5	Risk and hazard assessment of floods in urban areas	Flood, risk, area, assess, hazard, citi		26	
6	Model simulations of windstorms and water surges	Storm, wind, model, water, surg, simul		24	
7	The design of recovery projects in the city New Orleans	Recoveri, citi, design, build, project, orlean	Response and recovery (topic 7 and 8)	28	23.1%
8	Human responses to natural disasters and hurricanes	Disast, hurricane, respons, natur, human, state		23	
9	The health effects of hurricane Sandy on population	Health, sandi, resid, year, affect, popul	Health impacts (topic 9)	26	11.8%

**Fig 4:**
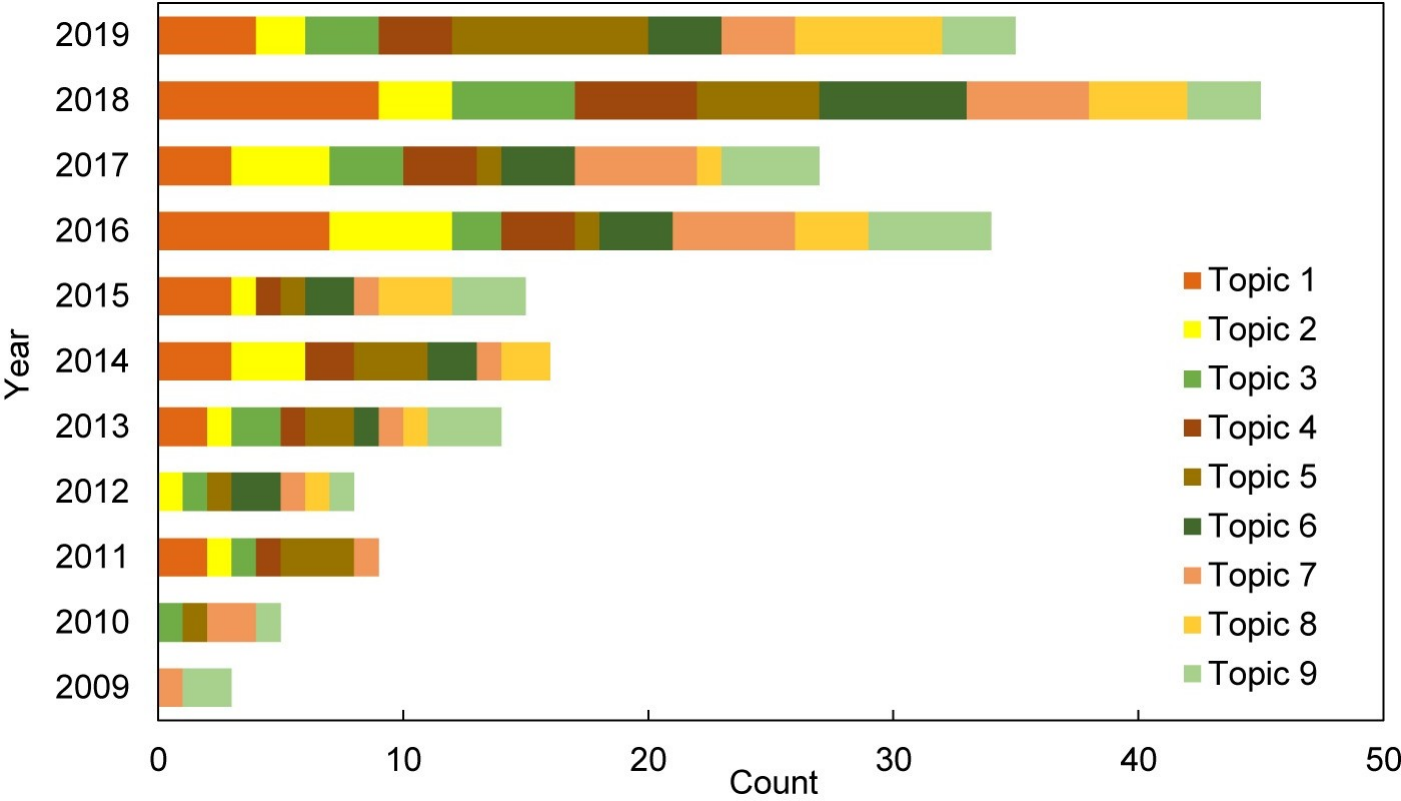
Annual distribution of topics for publications between 2009 and 2019.

For literature published in the same year, they cover diverse topics despite the small number of publications (Fig 4). At least six topics were covered in annual publications starting from 2011. 2013 was the first year that included all topics. But damages of extreme wind events and health impacts (topic 3 and topic 9) were missing in 2014, and only health impacts made their comeback in 2015. The past four years (2016–2019) included all the nine topics. Considering the cumulative total number of studies, we find that vulnerability and adaptation in the background of climate change (topic 1) have been the focus of studies in the past five years (from 2015 to 2019). The risk and hazard assessment of floods in cities (topic 5) has become the second most popular topic since 2018.

Some of these nine topics are intrinsically linked together, and they reflect a segment that contributes to successful adaptation. We further categorized the nine topics into the following four aspects: 1) vulnerability and resilience of cities, 2) damage evaluation, 3) response and recovery, and 4) health impacts. The evaluation of urban vulnerability is the starting point of designing adaptation measures. Because it examines the necessity to take actions and detects the most vulnerable parts. The ultimate goal of adaptation is to reduce vulnerability and increase resilience. Decision-makers can generate a more concrete idea about what negative consequences wind disasters have on cities by evaluating damages. The results are useful for cost assessment in pre-feasibility studies for adaptation projects. Health impacts are singled out to differentiate impacts on objects and impacts on humans, as the latter cannot be measured directly in monetary terms. The response and recovery of cities will most directly reveal how cities adapt to wind disasters, including cities’ post-disaster strengthening projects. Two categories directly related to the design of urban adaptation measures are the vulnerability of cities and reconstruction projects after wind disasters, which include 55 publications and 51 publications, respectively. Damage evaluation contains the most topics, and the number of publications under this aspect amounts to 90, indicating that current scientific studies are more focused on modeling, identifying, and assessing actual or possible consequences and damages. Studies related to health impacts are limited and contain the least number of articles. It indicates that health impacts are not yet well emphasized and require further attention.

#### Vulnerability and resilience of cities

Coastal areas are becoming more prone to wind-related storms and floods in the context of climate change [53–55]. The concentration of populations and properties [56], the dependence on coastal agriculture [57], the inequalities embedded in the socioeconomic system [58], and the reduction in areas of the ecosystem increase the vulnerability of coastal cities [59]. Besides, coastal storms and floods, along with resulted secondary hazards, are causing injuries and mental problems and increasing health vulnerability [60].

Ghaffarian et al. recognized vulnerability and resilience as two crucial dimensions in pre-disaster conditions for urban disaster management [61]. By establishing a vulnerability index to wind-related disasters at the city level, more vulnerable sectors can be identified, and this will tremendously help with planning and mitigation [56, 62–64]. It is crucial when cities have limited resources and need to allocate them effectively. The vulnerability index can also be established for specific sectors and specific disasters, e.g., Mallari et al. formed a vulnerability index for the agriculture sector against typhoons [65], and Martínez Méndez et al. made one for water infrastructures against hurricanes [66]. Likewise, scientists built frameworks for assessing the resilience of cities [67, 68] and for exploring important factors that contribute to the city’s resilience [69, 70]. McMillen et al. further discussed the application of resilience indicators in practical management operations either at the community or the city level [71].

Ecosystems play an important role in reducing vulnerabilities to climate change and hydrometeorological disasters [72, 73]. Meanwhile, community-based green practices such as gardens, are viewed as vital parts of the recovery processes after hurricanes [52, 74]. However, the development of urban areas and the destruction of hurricanes have led to less protection of ecosystems in cities [75–77]. Therefore, regulations and policies should are demanded to strengthen the resilience of urban ecosystems [78]. Infrastructures in coastal areas are exposed to greater risks of cyclonic storms [79], and economic damage of infrastructures induced by hurricanes is expected to increase due to climate change [80]. Hence, adaptation measures such as improving construction standards are in urgent need [81].

Although adaptation measures can bring about economic benefits and reduce a city’s vulnerability [82], they have the potential to cause negative effects on social capitals [83].Therefore, it is of vital significance to explore the optimal adaptation measures [84] and decide when to implement them in a structured way [85]. In coastal conditions, they should be flexible and diverse enough to cope with more powerful storms and floods in the future [86]. Scientists suggested scenario planning as an effective tool in ensuring flexible adaptation in given conditions [87]. Solecki et al. proposed a risk management framework that allows flexible changes of management patterns according to disasters [88]. Nevertheless, resilience-building still has to face multiple challenges that come from governance, finance, etc [89]. Besides, high-level adaptation actions are not yet in place [90].

To summarize, the first two topics discussed the vulnerability and resilience of coastal areas in the background of climate change and how to identify vulnerable parts by establishing an index. Ecosystem-based measures are highlighted to help develop social resilience and adaptation measures are suggested to be implemented based on scientific frameworks.

#### Damage evaluation

Topic 3, 4, 5, and 6 are related to the damages of wind-related disasters. The integration of urbanization, climate change, and storm surges are causing damages to cities [91]. The wind could either cause damages directly [92, 93] or induce secondary disasters such as snowfalls, extreme precipitations, and landslides [94–96]. Development of residential areas and migration to risky zones have increased exposure to tropical cyclones [97, 98]. Cumulative damages are expected to increase due to the rise of sea level and increase in the frequency of storms [99]. False psychological perception of extreme events can result in more severe suffering of damages [100]. The African-American community, women, and the elderly are reported to be the most affected demographic groups by hurricanes and extreme events [101, 102].

Floods along with wind-related storms are becoming principal hazards to cities [103, 104] by primarily causing damages to building structures [105]. Factors such as climate change, tropical cyclones, rainfall, and land use types are often taken into account when assessing current or future risks of floods [106–109]. Flood mapping is the main method used to present the spatial distribution of flooding risks. Based on the definition of risk, researchers usually develop the city-level flood risk index from a vulnerability and hazard perspective [110–112]. However, mapping without indicators is also possible. For example, Peng et al. mapped the flood extent for Houston and Lumberton with spectral reflectance images from satellites [113]. Apart from flood risks, there are also multi-hazard assessments that incorporate floods with other risks such as heat waves [114]. Further studies could be conducted by making use of the results from flood mapping. For example, flood risk mapping and social indicators are combined to examine the most exposed population [115]. Another example is that flood mapping is used to study the relationship between flood and land subsidence [116].

Damages on the systems, such as the traffic system, in cities are among the points of attention. Takayasu et al. generally introduced the simulation of road traffic against disasters such as cyclones [117]. Kontou et al. [118] and Zhong et al. [119] discussed impacts of hurricanes on the road traffic and traveling time in NYC with the help of big data derived from the movement of vehicles. Donovan et al. quantified the resilience of the traffic system in the city and demonstrated the severe traffic jam after Hurricane Sandy [120] while Zhu et al. modeled the recovery patterns of the traffic system after Sandy struck the city [121]. Some articles studied the resilience and fragility of the electric power system [122, 123]. In addition to single network analysis, articles were combing two or more networks to stress the interdependency of systems and cascading damages of disasters [124, 125].

The overall impacts resulted from wind-related disasters, such as typhoons, dust storms, and sandstorms could also be assessed by models [126, 127], regardless of poor data availability [128]. Apart from estimating the consequential impacts, models are useful in simulating and forecasting storm surges. Hurricane and cyclone-induced storm surges will increase the destructions of wind disasters [129]. When combined with sea-level rise, storm surges contribute to a dramatic increase in frequencies of extreme floods that threaten the security of the city [130]. Simulations with hydrodynamic models have focused on storm surges [131], and the resulting extreme waves [132, 133], water level increments, and flood events [134, 135]. These simulations play an important role in depicting and informing the risks that cities are facing, and thus are of great help in preparing cities for potential disasters through adaptation [134]. Additionally, model simulation is useful in better designing or choosing adaptation measures by simulating functions of controlling processes [136] and effects of adaptive actions [137]. The model forecasting range from indicators such as wind speed [138] and water level [139] to events such as dust storms [140], cyclones [141], and floods [142]. Forecasting is indispensable to alarm approaching disasters, and it allows more time for people to evacuate from dangerous zones, thus avoiding unnecessary losses of human lives.

In brief, both direct damages from wind disasters and indirect damages from secondary events such as storm surges and floods were covered. Network analysis was performed to track the performance of urban systems, especially the traffic system. Models have been developed and applied in assessing risks, simulating impacts, and forecasting future disasters to support adaptation decisions in a visualized way.

#### Response and recovery

Resettlement of impacted population and rebuilding of houses are the foci during the recovery period after tropical storms, hurricanes, and related floods. There are articles introducing the modification of houses [143], the inadequate support from local governments [144] and incompatibility of supply and demand [145], interactions of stakeholders during the processes [146], as well as the necessity to adapt recovery projects to local conditions [147]. Despite the urgent need for resettling after wind hazards, scientists noticed the environmental impacts of temporary housing during the recovery period [148]. The idea of sustainability is incorporated in the urban post-disaster recovery projects [149], with an emphasis on structural design [150] and green infrastructure projects [151, 152]. Hoeferlin promoted sustainability by reusing old buildings instead of demolishing them in New Orleans after Hurricane Katrina [153]. Articles sorted out hurricanes that had struck the city and the city’s development of adaptation [154], especially for Hurricane Katrina and the reconstruction period after it [155, 156]. The city was faced with difficulties attracting outflowed businesses after the disaster [157]. The factors that influence businesses to make recovery decisions were studied in NYC after Hurricane Sandy [158]. Apart from resettlement, other post-disaster actions, such as responses to Cholera after Cyclone Kenneth [159], and plans for improving water security and sanitation after Typhoon Haiyan [160], were also mentioned.

The topic of human responses covers several types of measures for cities and their residents to respond to wind hazards, especially to hurricanes. First, cities respond to hazards through evaluating risks, reducing vulnerabilities, and building resilience [161–165]. Second, estimates on total death during or after hurricanes are made to help provide adequate support to vulnerable populations [166, 167]. Finally, humans respond to wind disasters by evacuation [168] or by resettling [169]. Evidence implies that humans have a tendency to settle in disaster-prone areas [170], therefore research on human mobility is important for planning evacuations. Studies have shown that human mobility is resilient to wind hazards of certain intensity [171], and it complies with the power-law [171–173]. Data derived from social media such as Twitter is used for analyzing mobility patterns [171, 172, 174] and for evaluating economic damages [175]. Research contents of articles under this topic are closely linked to those under topic 7 which discussed the reconstruction of cities [176–178] and recovery of markets [179] since respondence can often occur after disasters.

Topic 7 and 8 reveal that households and businesses usually respond to wind disasters by evacuation and resettlement. Human mobility patterns are studied to support the making of evacuation plans. There are also studies working on factors that influence resettlement decisions to assist cities in reconstruction and attracting households and businesses back in the recovery process.

#### Health impacts

Scientists have identified that the elderly are more vulnerable to climate-related stressors [180], though they tend to be better prepared for disasters than young people [181]. A lot of articles are trying to estimate the health risks of the older population [182] and their vulnerability [180] when exposed to natural disasters. Some articles capture mental diseases such as post-traumatic stress disorder (PTSD), depression, and stress after hurricanes [183, 184] A few of these articles focus on the elder people [185, 186]. West et al. [187], Gruebner et al. [188], and Lee et al. [189] analyzed the geographical distribution and clusters of the population prone to negative health impacts of hurricanes while Gruebner et al. [190] studied the spatial patterns of vulnerability and resilience factors. Many articles studied social factors influencing the resilience of mental illness, such as being a member of a community [183] or having prior experience of natural disasters [191–193]. In addition to psychological health, there are articles discussing asthma among children and the effectiveness of interventions by providing consultations [194, 195]. Toner et al. summarized a series of adaptation actions to health impacts from Hurricane Sandy [196]. Specific adaptation measures such as community support for asthma after Hurricane Ike [187], stepped care and usual care for PTSD [197], health care for evacuees during Hurricane Katrina [198], and application of the Geographic Information System (GIS) in adapting emergency shelters to various hazards are covered in different articles [199]. Articles under this topic largely used methods such as literature research (e.g. [196]), telephone interview (e.g. [200]), face-to-face interview (e.g. [191]), and questionnaires (e.g. [184]).

Few articles studied the health impacts of wind disasters on humans. There is an obvious research bias towards psychological problems over physical harms resulted from hurricanes.

#### Topics related to cities

**Fig 5:**
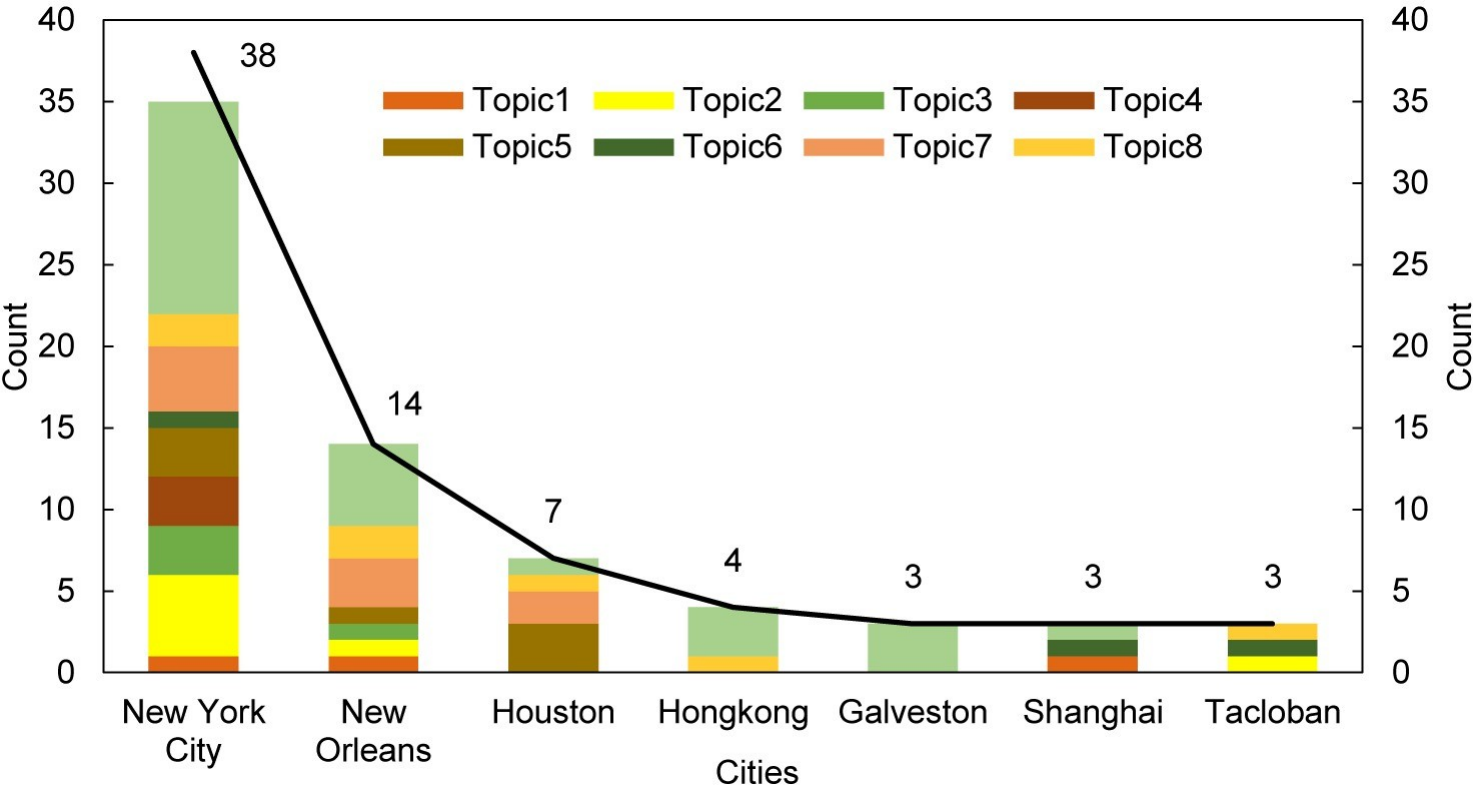
The distribution of topics within high-frequency cities.

We combined topics and cities to present topics that cities are focusing on (Fig 5). NYC was mentioned most frequently for 38 times, almost three times than that of New Orleans (14). Generally, the more often one city is covered in literature, the more diverse topics are related to that city. Yet, it is not the case for Shanghai and Tacloban. NYC is the only one connected to all nine topics, followed by New Orleans, which correlates to seven topics. The remaining cities are related to two to four topics except for Galveston, which relates to topic 9 only. The number of topics does not distribute evenly within cities. In addition to Galveston, the health impacts of wind-related disasters were the most discussed topic among NYC (13), New Orleans (5), and Hong Kong (3). It is also the topic that correlates with most cities and the topic of highest frequency (26) among nine cities. Although the topic of recovery processes also includes seven cities, its frequency (9) is much lower than that of health impacts. On average, one specific topic is only connected to half of the high-frequency cities.

### Analysis on adaptation measures

According to IPCC, adaptation measures are divided into three categories and ten subcategories [51]. We counted the number of adaptation measures for each category and subcategory (Table 2). Structural and physical adaptation measures (42.2%) are most frequently mentioned, followed by social measures (37.3%) and institutional measures (20.5%). The structural and physical category includes subcategories of engineered measures and built environment renovation (35), technological innovation (85), ecosystem-based actions (24), and services (37). The total number of measures amounts to 181. 160 adaptation measures belong to the social category, which was further grouped into subcategories such as educational (30), informational (101), and behavioral (29) measures. Institutional measures are composed of economic activities (25), laws and regulations (12), and government policies and programs (51). Informational action is the sub-category of the highest frequency, followed by technological innovation. Laws and regulations are the least favorite subcategory which has only 12 relevant adaptation measures.

**Table 2:** Number of adaptation measures grouped by categories and subcategories in the literature corpus.

Category	Subcategory	Number of Documents	Number of Documents within Category
**Structural and physical**	Engineered and built environment	35	181
	Technological	85	
	Ecosystem	24	
	Services	37	
**Social**	Educational	30	160
	Informational	101	
	Behavioral	29	
**Institutional**	Economic	25	88
	Laws and regulations	12	
	Gov. policies and programs	51	

**Fig 6:**
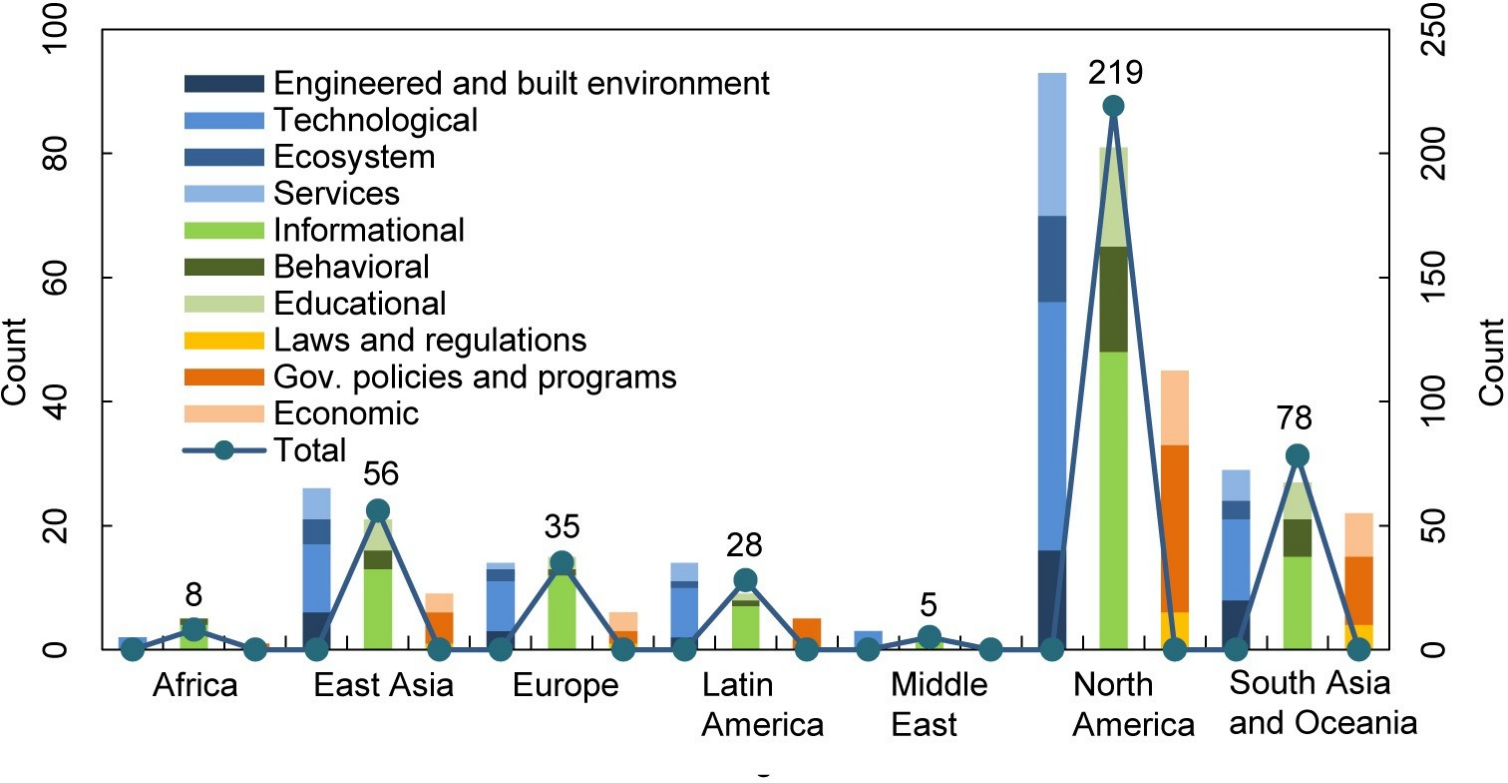
The total amount and distribution of different adaptation measures in seven regions.

Due to the extremely small number of adaptation measures connected with the specific city, we counted the number of different types of adaptation measures according to seven regions to which cities belong (Fig 6). The total number of adaptation measures varies greatly between these regions. It ranges from 219 in North America (51%) to just 5 in the Middle East (1.2%). Cities located in North America and Asia are more positively related to adaptation to wind hazards while those in Africa and the Middle East are in the initial stages of studying adaptation measures. Structural and physical adaptation measures are the most adopted types in East Asia, Latin America, Middle East, North America, and South Asia & Oceania, in compliance with the statistical results throughout all regions. In Africa and Europe, social measures exceed structural and physical ones. All ten subcategories of adaptation measures are included in studies in East Asia, Europe, North America, and South Asia & Oceania. Adaptation measures in Africa cover four subcategories while the Middle East covers two. Technological innovations and informational adaptation are the only two types studied by cities in all regions. Technological innovations dominate structural and physical measures in all regions, the percentage is 100% in Africa and the Middle East, 42.3% in East Asia, 57.1% in Europe and Latin America, 43% in North America, and 44.8% in South Asia and Oceania. Informational adaptation is currently prevailing among social measures. It accounts for 80% in Africa and Europe, 61.9% in East Asia, 77.8% in Latin America, 100% in the Middle East, 59.3% in North America, and 55.6% in Asia and Oceania. Government policies and programs share the highest percentage among institutional measures in all regions except Europe (one less than economic ones) and the Middle East (none). The corresponding proportion within the category is 100% in Africa and Latin America, 55.6% in East Asia, 60% in North America, and 50% in South Asia and Oceania.

**Fig 7:**
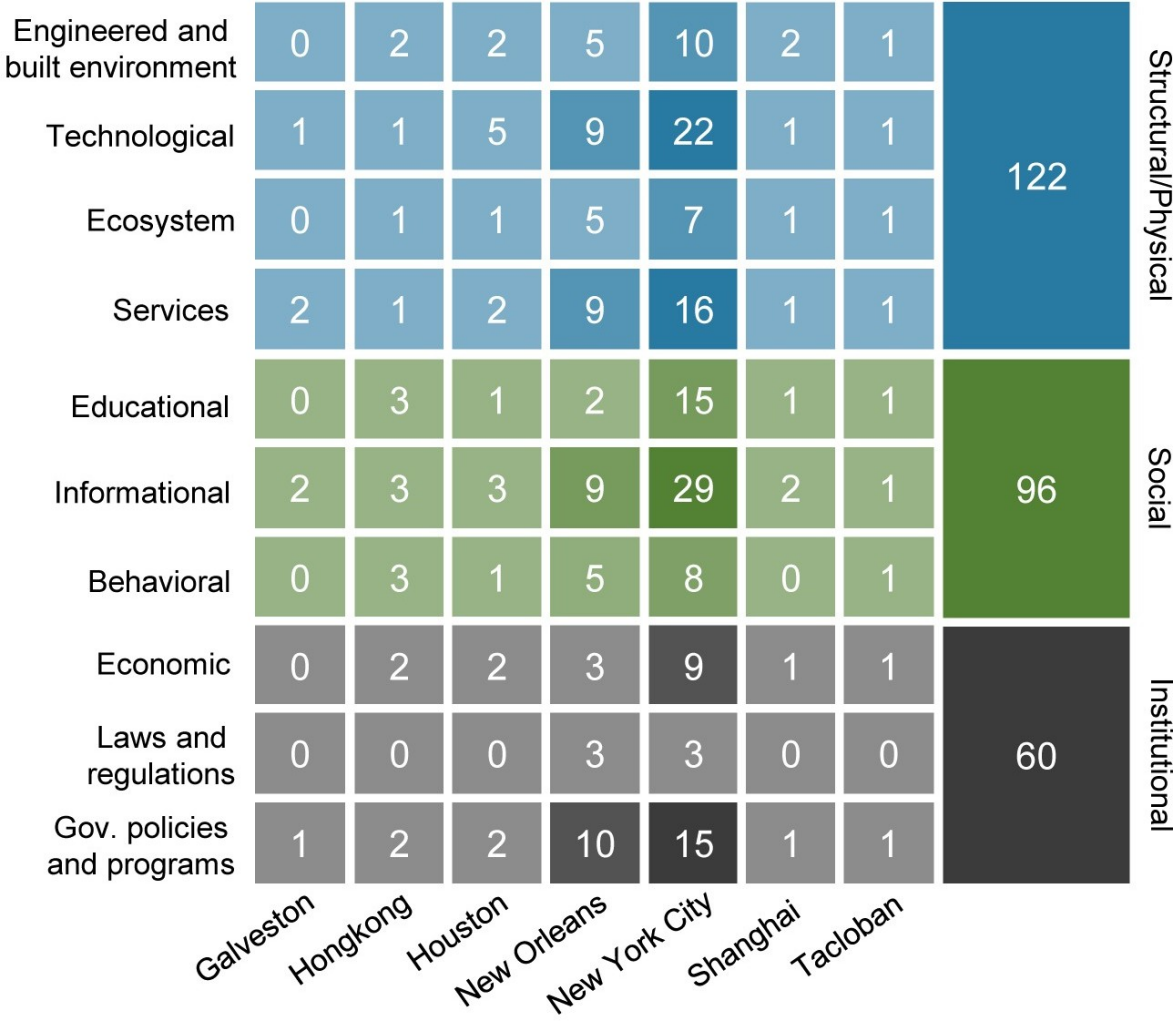
The total amount and distribution of different adaptation measures in seven high-frequency cities.

We extracted the statistics of adaptation measures based on categories and subcategories for the nine most frequently mentioned cities (Fig 7). Structural and physical measures remain the highest occurring measures, followed by social measures and institutional measures. Adaptation measures related to these nine cities are responsible for nearly 65% of the total, 67.4% for structural and physical ones, 60% for social ones, and 68.2% for institutional ones. NYC is most studied in terms of wind adaptation measures, with relevance to 134 measures. Therein, 55 are from structural/ physical ones, 52 from social ones, and 27 from institutional ones. The focus of wind adaptation measures in NYC is on informational measures, technological innovation, services, educational measures, and government policies and programs. New Orleans’s related adaptation measures are second only to NYC, a total of 60. Government policies and programs, technological innovation, services, and informational measures are the most popular ones for the city. NYC and New Orleans have far more numbers of adaptation measures than other cities. Meanwhile, they are the only two cities that have covered all types of adaptation measures. There are no related measures of laws and regulations found for Hong Kong, Houston, and Tacloban in the dataset. Apart from laws and regulations, Shanghai is not connected to behavioral measures as well. Galveston is found to have the least subcategories of adaptation measures in the dataset.

## Conclusion

We reviewed open access literature published in Web of Science and Scopus between 1900 and 2019 that discussed adaptation to wind-related disasters in cities. We conducted LDA and full-text review to discover major contents, focal cities, and popular adaptation measures in wind adaptation research. We paid special attention to climate change to examine whether it has been incorporated into wind disaster adaptation studies.

We find that wind adaptation studies experienced fluctuated growth in the past decade. Cities located in North America, East Asia, and South Asia are more often targeted in this field. This is probably due to the high concentrations of developed economies in these regions [201]. Also, they are associated with more diversified topics and adaptation measures. The literature most frequently mentioned NYC and New Orleans because they have experienced Hurricane Sandy [30] and Hurricane Katrina [202] respectively. It indicates that cities once suffered from deadly or costly wind disasters are more likely to become the objects of adaptation research.

The four major aspects covered by the studies are 1) the vulnerability and resilience of cities, 2) evaluations on damages, 3) response to disasters and recovery processes, and 4) health impacts of wind disasters. The necessity of studying natural hazards in the background of climate change has been recognized [203]. In this study, climate change is especially embedded in the first two aspects. It appears among the key root words in the largest topic set, along with coastal vulnerability and adaptation. This demonstrates that current research has noticed the impact of future climate change on urban vulnerability since it is an external stressor that enhances the destructive power of wind disasters. Cities located in coastal areas are of special concern because they are at the forefront of many hydrometeorological climatic hazards. Climate change could interact with urbanization processes and wind disaster climatology; thus, climate scenario is necessary for models which will allow scientists to obtain more accurate assessments on urban exposure and future damages. Wind disasters such as hurricanes cause abrupt consequences. But the impacts of climate change can be distant and moderate. Moreover, the latter often exacerbates before people realize them. In this case, adaptation measures will both defend short-term disasters and take precautions against long-term changes in a more sustainable approach.

Structural and physical adaptation measures are the most covered measures (181) in selected papers, followed by social measures (160) and institutional measures (88). We found distinct differences in the number of different adaptation subcategories. 101 measures are technological innovations, but only 12 measures are laws and regulations. Adaptation subcategories show diverse proportion distributions in cities in different regions. In general, scientists prefer measures that are more easily achieved through technological development, informational systems, and government policies and programs. The latest study by Du et al. shows that a combination of hard measures and soft measures can significantly reduce risks and improve economic efficiency [204]. Though current studies present bias towards hard measures, the hybrid development of both measures is taking shape. This calls for more attention to soft measures such as the making of laws and regulations.

Only 11.8% of the literature concentrates on the health impacts of wind disasters in this dataset. It is much less when compared to the literature discussing physical damages. This reveals the limited attention that is paid to health issues. Meanwhile, their discussions also lack links to climate change. Climate change is viewed as “the greatest global health opportunity of the 21^st^ century” by Lancet [205]. Incorporating it into wind adaptation not only makes causes of negative health impacts more comprehensive but also generates co-benefits by finding common solutions. Health adaptation to climate change is expected to be part of national plans in the future [206]. This potentially promotes wind adaptation even if a city has yet to realize that health problems that can stem from wind disasters.

Wind disasters, especially tropical storms, have attracted much attention. These disasters, along with their secondary hazards, are damaging urban systems such as the traffic system. Cities rely on all kinds of indices to evaluate their vulnerability and to identify the most demanding sectors. They have also noticed the necessity to include climate change while doing the evaluation. However, the physical and psychological impacts of wind disasters need more emphasis. NYC’s experience indicates that a catastrophic disaster can greatly motivate adaptation progress in the city. Cities are actively taking various measures in forecasting and evacuation, to minimize losses against disasters. Yet, hard measures belonging to structural and physical measures remain more popular in general.

Our review provides an insight into the literature regarding adaptation to wind-related hazards in cities within the open access area, where the public is provided with adequate scientific knowledge and information. However, this study still has the following limitations and needs further development. First, this study only depicts the full picture of wind disaster adaptation in global cities but fails to tell the research contents studied surrounding a specific city. Therefore, it is unable to offer concrete suggestions to designated cities. Besides, we are unable to show the dynamic changes of cities that have experienced wind disasters on a longer time scale due to the inclusion of very few numbers of articles published before 2010. Furthermore, we did not explore the characteristics of cities that have more adaptation measures. Research could improve by conducting a more detailed full-text analysis to establish actual relationships between cities and adaptation measures in the future. The matching of cities and the year in which disasters occurred as well as when the study was published could also be included in the future. By collecting socio-economic and meteorological data of cities, common features may be identified for cities that are more active in adapting to wind disasters.

## Supporting information

S1 Checklist PRISMA checklist. (DOC)

S1 Table List of publications and statistic results. (XLSX)

S2 Table List of cities and their frequencies. (XLSX)

S3 Table Quality assessment. (XLSX)

S1 Appendix The final search queries for Web of Science and Scopus. (DOCX)

S2 Appendix A templet for targeted information in the literature corpus. (DOCX)

## References

[1] Bloomberg M. A stronger, more resilient New York. New York: City of New York Mayor’s Office. 2013. Available: http://s-media.nyc.gov/agencies/sirr/SIRR_singles_Lo_res.pdf

[2] Yang Q, Gao R, Bai F, Li T, Tamura Y. Damage to buildings and structures due to recent devastating wind hazards in East Asia. Nat Hazards. 2018;92: 1321–1353. 10.1007/s11069-018-3253-8

[3] Zampieri NE, Pau S, Okamoto DK. The impact of Hurricane Michael on longleaf pine habitats in Florida. Scientific Reports. 2020;10: 8483. 10.1038/s41598-020-65436-9 32439960 PMC7242371

[4] Irfan FB, Pathan SA, Bhutta ZA, Abbasy ME, Elmoheen A, Elsaeidy AM, et al. Health system response and adaptation to the largest sandstorm in the Middle East. Disaster Med Public Health Prep. 2017;11: 227–238. 10.1017/dmp.2016.111 27539443

[5] D. Guha-Sapir. EM-DAT: The Emergency Events Datebase. [cited 5 Dec 2020]. Available: https://public.emdat.be/data

[6] NOAA National Centers for Environmental Information (NCEI) U.S. Billion-Dollar Weather and Climate Disasters (2020). 10.25921/STKW-7W73

[7] The Lancet Planetary Health. Hurricanes and architecture: adaptation to the destruction. The Lancet Planetary Health. 2018;2: e414. 10.1016/S2542-5196(18)30204-3 30318093

[8] Finlayson CM, D’Cruz R, Davidson N, Alder J, Cork S, Groot RD, et al. Ecosystems and human well-being: wetlands and water synthesis. Washington, DC: World Resources Institute; 2005.

[9] Stocker TF, Qin D, Plattner G-K, Tignor MMB, Allen SK, Boschung J, et al. Climate change 2013: The physical science basi: Working group I contribution to the fifth assessment report of the intergovernmental panel on climate change. Cambridge University Press; 2013.

[10] Stewart MG, Wang X, Willgoose GR. Direct and indirect cost-and-benefit assessment of climate adaptation strategies for housing for extreme wind events in Queensland. Nat Hazards Rev. 2014;15: 04014008. 10.1061/(ASCE)NH.1527-6996.0000136

[11] Stewart MG. Risk and economic viability of housing climate adaptation strategies for wind hazards in southeast Australia. Mitig Adapt Strateg Glob Change. 2015;20: 601–622. 10.1007/s11027-013-9510-y

[12] Atkinson RW, Kang S, Anderson HR, Mills IC, Walton HA. Epidemiological time series studies of PM _2.5_ and daily mortality and hospital admissions: a systematic review and meta-analysis. Thorax. 2014;69: 660–665. 10.1136/thoraxjnl-2013-204492 24706041 PMC4078677

[13] Hurricane and tropical storm watches, warnings, advisories and outlooks. NOAA’s National Weather Service; [cited 1 Dec 2020]. Available: https://www.weather.gov/safety/hurricane-ww

[14] Shanghai Meteorological Bureau, CMA. Overview of Shanghai multi-hazard early warning system and the role of meteorological services. 2010. Available: https://www.wmo.int/pages/prog/drr/events/MHEWSCostaRica/Docs/Session%202/Shanghai/Shanghai_MHEWS_CostaRica.pdf

[15] Emanuel KA. The dependence of hurricane intensity on climate. Nature. 1987;326: 483–485. 10.1038/326483a0

[16] McGranahan G, Balk D, Anderson B. The rising tide: Assessing the risks of climate change and human settlements in low elevation coastal zones. Environment and Urbanization. 2007;19: 17–37. 10.1177/0956247807076960

[17] Woodruff JD, Irish JL, Camargo SJ. Coastal flooding by tropical cyclones and sea-level rise. Nature. 2013;504: 44–52. 10.1038/nature12855 24305147

[18] Trenberth K. Uncertainty in Hurricanes and Global Warming. Science. 2005;308: 1753–1754. 10.1126/science.1112551 15961661

[19] Lin N, Emanuel K, Oppenheimer M, Vanmarcke E. Physically based assessment of hurricane surge threat under climate change. Nature Clim Change. 2012;2: 462–467. 10.1038/nclimate1389

[20] Mendelsohn R, Emanuel K, Chonabayashi S, Bakkensen L. The impact of climate change on global tropical cyclone damage. Nature Climate Change. 2012;2: 205–209. 10.1038/nclimate1357

[21] Landsea CW, Harper BA, Hoarau K, Knaff JA. Can we detect trends in xxtreme tropical cyclones? Science. 2006;313: 452–454. 10.1126/science.1128448 16873634

[22] Knutson TR, McBride JL, Chan J, Emanuel K, Holland G, Landsea C, et al. Tropical cyclones and climate change. Nat Geosci. 2010;3: 157–163. 10.1038/ngeo779

[23] Tamura Y. Wind-induced damage to buildings and disaster risk reduction. Taipei, Taiwan; 2009.

[24] Issa A, Ramadugu K, Mulay P, Hamilton J, Siegel V, Harrison C, et al. Deaths related to Hurricane Irma—Florida, Georgia, and North Carolina, September 4–October 10, 2017. MMWR Morb Mortal Wkly Rep. 2018;67: 829–832. 10.15585/mmwr.mm6730a5 30070979 PMC6072056

[25] Tan C, Fang W. Mapping the wind hazard of global tropical cyclones with parametric wind field models by considering the effects of local factors. Int J Disaster Risk Sci. 2018;9: 86–99. 10.1007/s13753-018-0161-1

[26] CDP Open Data Portal. [cited 5 Dec 2020]. Available: https://data.cdp.net/browse?category=Climate+Hazards

[27] Droste AM, Steeneveld GJ, Holtslag AAM. Introducing the urban wind island effect. Environ Res Lett. 2018;13: 094007. 10.1088/1748-9326/aad8ef

[28] Blocken B, Carmeliet J, Stathopoulos T. CFD evaluation of wind speed conditions in passages between parallel buildings—effect of wall-function roughness modifications for the atmospheric boundary layer flow. J Wind Eng Ind Aerodyn. 2007;95: 941–962. 10.1016/j.jweia.2007.01.013

[29] Dutt AJ. Wind flow in an urban environment. Environ Monit Assess. 1991;19: 495–506. 10.1007/BF00401336 24233964

[30] Rosenzweig C, Solecki W. Hurricane Sandy and adaptation pathways in New York: Lessons from a first-responder city. Global Environmental Change. 2014;28: 395–408. 10.1016/j.gloenvcha.2014.05.003

[31] Godschalk DR. Urban hazard mitigation: Creating resilient cities. Nat Hazards Rev. 2003;4: 136–143. 10.1061/(ASCE)1527-6988(2003)4:3(136)

[32] Ford JD, Berrang-Ford L, Paterson J. A systematic review of observed climate change adaptation in developed nations: A letter. Clim Change. 2011;106: 327–336. 10.1007/s10584-011-0045-5

[33] Araos M, Berrang-Ford L, Ford JD, Austin SE, Biesbroek R, Lesnikowski A. Climate change adaptation planning in large cities: A systematic global assessment. Environ Sci Policy. 2016;66: 375–382. 10.1016/j.envsci.2016.06.009

[34] Georgeson L, Maslin M, Poessinouw M, Howard S. Adaptation responses to climate change differ between global megacities. Nature Clim Change. 2016;6: 584–588. 10.1038/nclimate2944

[35] Whitmarsh L. Are flood victims more concerned about climate change than other people? The role of direct experience in risk perception and behavioural response. J Risk Res. 2008;11: 351–374. 10.1080/13669870701552235

[36] Laakso M, Welling P, Bukvova H, Nyman L, Björk B-C, Hedlund T. The development of open access journal publishing from 1993 to 2009. Hermes-Lima M, editor. PLoS ONE. 2011;6: e20961. 10.1371/journal.pone.0020961 21695139 PMC3113847

[37] Piwowar H, Priem J, Larivière V, Alperin JP, Matthias L, Norlander B, et al. The state of OA: A large-scale analysis of the prevalence and impact of open access articles. PeerJ. 2018;6: e4375. 10.7717/peerj.4375 29456894 PMC5815332

[38] Wang X, Liu C, Mao W, Fang Z. The open access advantage considering citation, article usage and social media attention. Scientometrics. 2015;103: 555–564. 10.1007/s11192-015-1547-0

[39] Vogel B, Henstra D. Studying local climate adaptation: A heuristic research framework for comparative policy analysis. Glob Environ Change. 2015;31: 110–120. 10.1016/j.gloenvcha.2015.01.001

[40] Rosenzweig C, Solecki W, Romero-Lankao P, Mehrotra S, Dhakal S. Climate change and cities: Second assessment report of the urban climate change research network. Cambridge University Press; 2018.

[41] Mongeon P, Paul-Hus A. The journal coverage of Web of Science and Scopus: A comparative analysis. Scientometrics. 2016;106: 213–228. 10.1007/s11192-015-1765-5

[42] Gavel Y, Iselid L. Web of Science and Scopus: A journal title overlap study. Online Inf Rev. 2008;32: 8–21. 10.1108/14684520810865958

[43] Vera-Baceta M-A, Thelwall M, Kousha K. Web of Science and Scopus language coverage. Scientometrics. 2019;121: 1803–1813. 10.1007/s11192-019-03264-z

[44] Falagas ME, Pitsouni EI, Malietzis GA, Pappas G. Comparison of PubMed, Scopus, Web of Science, and Google Scholar: Strengths and weaknesses. FASEB j. 2008;22: 338–342. 10.1096/fj.07-9492LSF 17884971

[45] Jelodar H, Wang Y, Yuan C, Feng X, Jiang X, Li Y, et al. Latent dirichlet allocation (LDA) and topic modeling: Models, applications, a survey. Multimed Tools Appl. 2019;78: 15169–15211. 10.1007/s11042-018-6894-4

[46] Blei DM, NG AY, Jordan MI. Latent dirichlet allocation. J Mach Learn Res. 2003;3: 993–1022.

[47] Lamb WF, Callaghan MW, Creutzig F, Khosla R, Minx JC. The literature landscape on 1.5°C climate change and cities. Current Opinion in Environmental Sustainability. 2018;30: 26–34. 10.1016/j.cosust.2018.02.008

[48] Lamb WF, Creutzig F, Callaghan MW, Minx JC. Learning about urban climate solutions from case studies. Nat Clim Chang. 2019;9: 279–287. 10.1038/s41558-019-0440-x

[49] Wang Y-X, Zhang Y-J. Nonnegative matrix factorization: A comprehensive review. IEEE Trans Knowl Data Eng. 2013;25: 1336–1353. 10.1109/TKDE.2012.51

[50] McCallum A, Mimno DM, Wallach HM. Rethinking LDA: Why priors matter. In: Bengio Y, Schuurmans D, Lafferty JD, Williams CKI, Culotta A, editors. Advances in neural information processing systems 22. 2009. pp. 1973–1981.

[51] Climate change 2014: synthesis report. Contribution of working groups I, II and III to the fifth assessment report of the intergovernmental panel on climate change. Geneva, Switzerland; 2015.

[52] Chan J, DuBois B, Tidball KG. Refuges of local resilience: Community gardens in post-Sandy New York City. Urban For Urban Green. 2015;14: 625–635. 10.1016/j.ufug.2015.06.005

[53] Amante CJ. Uncertain seas: Probabilistic modeling of future coastal flood zones. Int J Geogr Inf Sci. 2019;33: 2188–2217. 10.1080/13658816.2019.1635253

[54] Lontone A, Ernšteins R, Lagzdiņa Ē, Vanaga M, Šteinberga Z. Flood risk governance and communication development for coastal municipalities: Information and education, participation and practise self-experience. 2017. pp. 373–385. 10.5593/sgem2017/52

[55] Gopalakrishnan T, Hasan MK, Haque ATMS, Jayasinghe SL, Kumar L. Sustainability of coastal agriculture under climate change. Sustainability. 2019;11: 7200. 10.3390/su11247200

[56] Lee Y-J. A synthesized biophysical and social vulnerability assessment for Taiwan. IOP Conf Ser: Earth Environ Sci. 2017;94: 012161. 10.1088/1755-1315/94/1/012161

[57] Ayeb-Karlsson S, van der Geest K, Ahmed I, Huq S, Warner K. A people‐centred perspective on climate change, environmental stress, and livelihood resilience in Bangladesh. Sustain Sci. 2016;11: 679–694. 10.1007/s11625-016-0379-z 30174739 PMC6106091

[58] Chuang W-C, Eason T, Garmestani A, Roberts C. Impact of Hurricane Katrina on the coastal systems of southern Louisiana. Front Environ Sci. 2019;7: 68. 10.3389/fenvs.2019.00068 32601598 PMC7321930

[59] Hopper T, Meixler MS. Modeling coastal vulnerability through space and time. PLoS ONE. 2016;11: e0163495. 10.1371/journal.pone.0163495 27732674 PMC5061324

[60] Lane K, Charles-Guzman K, Wheeler K, Abid Z, Graber N, Matte T. Health effects of coastal storms and flooding in urban areas: A review and vulnerability assessment. J Environ Public Health. 2013;2013: 1–13. 10.1155/2013/913064 23818911 PMC3683478

[61] Ghaffarian S, Kerle N, Filatova T. Remote sensing-based proxies for urban disaster risk management and resilience: A Review. Remote Sens. 2018;10: 1760. 10.3390/rs10111760

[62] Kim HG, Lee DK, Jung H, Kil S-H, Park JH, Park C, et al. Finding key vulnerable areas by a climate change vulnerability assessment. Nat Hazards. 2016;81: 1683–1732. 10.1007/s11069-016-2151-1

[63] Estember RD, Abiog MCM. Vulnerability assessment of Pangasinan province to typhoons, floods and landslides. Bandung, Indonesia; 2018. p. 020060. 10.1063/1.5080873

[64] Serafim MB, Bonetti J. Vulnerability of Santa Catarina Beaches to coastal erosion and flooding: A methodological approach based on a multicriterial index. Quat Environ Geosci. 2017;8: 36–54. 10.5380/abequa.v8i2.47281

[65] Mallari Ezra CA. Climate change vulnerability assessment in the agriculture sector: Typhoon Santi experience. Procedia Soc Behav Sci. 2016;216: 440–451. 10.1016/j.sbspro.2015.12.058

[66] Anita Martínez Méndez, Oscar Frausto Martínez, Lourdes Castillo Villanueva, José Manuel Camacho Sanabria. Index of Potable Water Infrastructure Resilience Facing Hurricanes in Coastal Cities. REVGEO. 2018;3: 339–365. 10.15359/rgac.61-3.17

[67] Sharifi A, Yamagata Y. Resilient urban planning: Major principles and criteria. Energy Procedia. 2014;61: 1491–1495. 10.1016/j.egypro.2014.12.154

[68] Frausto O, Vazquez A, Arroyo L, Castillo L, Hernández ML. Hurricane resilience indicators in mexican caribbean coastal cities. Int J SAFE. 2016;6: 755–763. 10.2495/SAFE-V6-N4-755-763

[69] Simonovic SP, Peck A. Dynamic Resilience to Climate Change Caused Natural Disasters in Coastal Megacities Quantification Framework. BJECC. 2013;3: 378–401. 10.9734/BJECC/2013/2504

[70] Graham L, Debucquoy W, Anguelovski I. The influence of urban development dynamics on community resilience practice in New York City after Superstorm Sandy: Experiences from the Lower East Side and the Rockaways. Glob Environ Change. 2016;40: 112–124. 10.1016/j.gloenvcha.2016.07.001

[71] McMillen H, Campbell L, Svendsen E, Reynolds R. Recognizing stewardship practices as indicators of social resilience: In living memorials and in a community garden. Sustainability. 2016;8: 775. 10.3390/su8080775

[72] Sajjad M, Li Y, Tang Z, Cao L, Liu X. Assessing hazard vulnerability, habitat conservation, and restoration for the enhancement of mainland China’s coastal resilience. Earth’s Future. 2018;6: 326–338. 10.1002/2017EF000676

[73] Pérez-Maqueo O, Martínez M, Sánchez-Barradas F, Kolb M. Assessing nature-based coastal protection against disasters derived from extreme hydrometeorological events in Mexico. Sustainability. 2018;10: 1317. 10.3390/su10051317

[74] Tidball KG. Seeing the forest for the trees: Hybridity and social-ecological symbols, rituals and resilience in postdisaster contexts. E&S. 2014;19: art25. 10.5751/ES-06903-190425

[75] Mosley S. Coastal cities and environmental change. Environ Hist Camb. 2014;20: 517–533. 10.3197/096734014X14091313617280

[76] Otero FG. Methodology for the monitoring of the mangrove recovery in the mouth of the San Cristobal River. Revista Cubana de Ciencias Forestales. 2018;6: 240–256.

[77] Bures R, Kanapaux W. Historical regimes and social indicators of resilience in an urban system: the case of Charleston, South Carolina. E&S. 2011;16: art16. 10.5751/ES-04293-160416

[78] Gotham KF, Campanella R. Coupled vulnerability and resilience: the dynamics of cross-scale interactions in post-Katrina New Orleans. E&S. 2011;16: art12. 10.5751/ES-04292-160312

[79] Rahman S, Rahman MA. Climate extremes and challenges to infrastructure development in coastal cities in Bangladesh. Weather Clim Extremes. 2015;7: 96–108. 10.1016/j.wace.2014.07.004

[80] Cui W, Caracoglia L. Exploring hurricane wind speed along US Atlantic coast in warming climate and effects on predictions of structural damage and intervention costs. Eng Struct. 2016;122: 209–225. 10.1016/j.engstruct.2016.05.003

[81] Wilbanks TJ, Fernandez S, Backus G, Garcia P, Jonietz KK, Kirshen PH, et al. Climate change and infrastructure, urban systems, and vulnerabilities: Technical report for the U.S. Department of Energy in support of the national climate assessment. Washington, DC: Island Press; 2014. 10.5822/978-1-61091-556-4

[82] Hallegatte S, Ranger N, Mestre O, Dumas P, Corfee-Morlot J, Herweijer C, et al. Assessing climate change impacts, sea level rise and storm surge risk in port cities: a case study on Copenhagen. Climatic Change. 2011;104: 113–137. 10.1007/s10584-010-9978-3

[83] Eadie P, Su Y. Post-disaster social capital: trust, equity, bayanihan and Typhoon Yolanda. Disaster Prev and Management. 2018;27: 334–345. 10.1108/DPM-02-2018-0060

[84] Raza T. Localizing disaster risk reduction and climate change adaptation in planners’ and decision makers’ agenda: Technical comprehensive model, Quezon City, Philippines. Procedia Engineering. 2018;212: 1311–1318. 10.1016/j.proeng.2018.01.169

[85] Ke Q, Haasnoot M, Hoogvliet M. Exploring adaptation pathways in terms of flood risk management at a city scale–a case study for Shanghai city. Lang M, Klijn F, Samuels P, editors. E3S Web Conf. 2016;7: 21002. 10.1051/e3sconf/20160721002

[86] Williams S, Ismail N. Climate change, coastal vulnerability and the need for adaptation alternatives: Planning and design examples from Egypt and the USA. J Mar Sci Eng. 2015;3: 591–606. 10.3390/jmse3030591

[87] De Lotto R, Gazzola Cecilia Morelli di Popolo V, Maria Venco E. From resilience to flexibility: Urban scenario to reduce hazard. Int J SDP. 2017;12: 789–799. 10.2495/SDP-V12-N4-789-799

[88] Solecki W, Pelling M, Garschagen M. Transitions between risk management regimes in cities. E&S. 2017;22: art38. 10.5751/ES-09102-220238

[89] Kiddle G, McEvoy D, Mitchell D, Jones P, Mecartney S. Unpacking the pacific urban agenda: Resilience challenges and opportunities. Sustainability. 2017;9: 1878. 10.3390/su9101878

[90] Magee AD, Verdon-Kidd DC, Kiem AS, Royle SA. Tropical cyclone perceptions, impacts and adaptation in the Southwest Pacific: An urban perspective from Fiji, Vanuatu and Tonga. Nat Hazards Earth Syst Sci. 2016;16: 1091–1105. 10.5194/nhess-16-1091-2016

[91] Lin N, Shullman E. Dealing with hurricane surge flooding in a changing environment: part I. Risk assessment considering storm climatology change, sea level rise, and coastal development. Stoch Environ Res Risk Assess. 2017;31: 2379–2400. 10.1007/s00477-016-1377-5

[92] Tavakkolifard A, Ghasemieh H, Samani AAN, Mashhadi N. Determining the risk of sand transportation to residential areas around Kashan Erg using anemometry data analysis. Desert. 2013;10: 163–172.

[93] Grøntoft T. Observed recent change in climate and potential for decay of Norwegian wood structures. Climate. 2019;7: 33. 10.3390/cli7020033

[94] Baijnath-Rodino JA, Duguay CR. Historical spatiotemporal trends in snowfall extremes over the Canadian domain of the Great Lakes Basin. Adv Meteorol. 2018;2018: 1–20. 10.1155/2018/5404123

[95] Borja Baeza RC, Alcántara Ayala I. Mass movement processes and associated risks in Zacapoaxtla, Puebla. Investigaciones Geográficas. 2012; 7. 10.14350/rig.30197

[96] Song Y-K, Oh J, Son Y-J, Jung M. Evaluation of landslide susceptibility using scientific investigation and quantitative evaluation. IJDRBC. 2013;4: 1–10. 10.14257/ijdrbc.2013.4.01

[97] Freeman AC, Ashley WS. Changes in the US hurricane disaster landscape: the relationship between risk and exposure. Nat Hazards. 2017;88: 659–682. 10.1007/s11069-017-2885-4

[98] Klose CD. Evidence for higher tropical storm risks in Haiti due to increasing population density in hazard prone urban areas. Environ Res Lett. 2011;6: 044020. 10.1088/1748-9326/6/4/044020

[99] Small MJ, Xian S. A human-environmental network model for assessing coastal mitigation decisions informed by imperfect climate studies. Glob Environ Change. 2018;53: 137–145. 10.1016/j.gloenvcha.2018.09.006

[100] Dark SM. The unconscious mind rules in absentia. WIT Trans Ecol Environ. 2016;204: 599–610. 10.2495/SC160501

[101] Fussell E, Sastry N, VanLandingham M. Race, socioeconomic status, and return migration to New Orleans after Hurricane Katrina. Popul Environ. 2010;31: 20–42. 10.1007/s11111-009-0092-2 20440381 PMC2862006

[102] Reckien D, Petkova EP. Who is responsible for climate change adaptation? Environ Res Lett. 2019;14: 014010. 10.1088/1748-9326/aaf07a

[103] Juanzon JBP, Oreta AWC. An assessment on the effective preparedness and disaster response: The case of Santa Rosa City, Laguna. Procedia Engineering. 2018;212: 929–936. 10.1016/j.proeng.2018.01.120

[104] van Ree CCDF, Van MA, Heilemann K, Morris MW, Royet P, Zevenbergen C. FloodProBE: Technologies for improved safety of the built environment in relation to flood events. Environ Sci Policy. 2011;14: 874–883. 10.1016/j.envsci.2011.03.010

[105] Rözer V, Kreibich H, Schröter K, Müller M, Sairam N, Doss‐Gollin J, et al. Probabilistic models significantly reduce uncertainty in Hurricane Harvey pluvial flood loss estimates. Earth’s Future. 2019;7: 384–394. 10.1029/2018EF001074

[106] Czajkowski J, Villarini G, Montgomery M, Michel-Kerjan E, Goska R. Assessing current and future freshwater flood risk from North Atlantic tropical cyclones via insurance claims. Sci Rep. 2017;7: 41609. 10.1038/srep41609 28148952 PMC5288645

[107] Smith JB, Strzepek KM, Cardini J, Castaneda M, Holland J, Quiroz C, et al. Coping with climate variability and climate change in La Ceiba, Honduras. Clim Change. 2011;108: 457–470. 10.1007/s10584-011-0161-2

[108] Lian JJ, Xu K, Ma C. Joint impact of rainfall and tidal level on flood risk in a coastal city with a complex river network: a case study for Fuzhou city, China. Hydrol Earth Syst Sci Discuss. 2012;9: 7475–7505. 10.5194/hessd-9-7475-2012

[109] Yang D, Yang A, Qiu H, Zhou Y, Herrero H, Fu C-S, et al. A citizen-contributed GIS approach for evaluating the impacts of land use on Hurricane-Harvey-induced flooding in Houston area. Land. 2019;8: 25. 10.3390/land8020025

[110] Armenakis C, Nirupama N. Flood risk mapping for the city of Toronto. Procedia Econ Financ. 2014;18: 320–326. 10.1016/S2212-5671(14)00946-0

[111] Shi Y, Zhai G, Zhou S, Lu Y, Chen W, Deng J. How can cities respond to flood disaster risks under multi-scenario simulation? A case study of Xiamen, China. Int J Environ Res Public Health. 2019;16: 618. 10.3390/ijerph16040618 30791558 PMC6406649

[112] Zhang J, Chen Y. Risk assessment of flood disaster induced by typhoon rainstorms in Guangdong Province, China. Sustainability. 2019;11: 2738. 10.3390/su11102738

[113] Peng B, Meng Z, Huang Q, Wang C. Patch similarity convolutional neural network for urban flood extent mapping using bi-temporal satellite multispectral imagery. Remote Sens. 2019;11: 2492. 10.3390/rs11212492

[114] Depietri Y, Dahal K, McPhearson T. Multi-hazard risks in New York City. Nat Hazards Earth Syst Sci. 2018;18: 3363–3381. 10.5194/nhess-18-3363-2018

[115] de Sherbin A, Bardy G. Social vulnerability to floods in two coastal megacities: New York City and Mumbai. Vienna Yearb Popul Res. 2016;1: 131–165. 10.1553/populationyearbook2015s131

[116] Miller MM, Shirzaei M. Land subsidence in Houston correlated with flooding from Hurricane Harvey. Remote Sens Environ. 2019;225: 368–378. 10.1016/j.rse.2019.03.022

[117] Sousa M.Y.R., Hideki Takayasu, Misako Takayasu, Michael Harré, Jean-François Boilard, Joshin Murai, et al. Proceedings of the international conference on social modeling and simulation, plus econophysics colloquium 2014. Takayasu H, Ito N, Noda I, Takayasu M, editors. Cham: Springer Proceedings in Complexity; 2015. 10.1007/978-3-319-20591-5

[118] Kontou E, Murray-Tuite P, Wernstedt K. Duration of commute travel changes in the aftermath of Hurricane Sandy using accelerated failure time modeling. Transp Res Part A Policy Pract. 2017;100: 170–181. 10.1016/j.tra.2017.04.015

[119] Zhong RX, Xie XX, Luo JC, Pan TL, Lam WHK, Sumalee A. Modeling double time-scale travel time processes with application to assessing the resilience of transportation systems. Transport Res Part B Meth. 2020;132: 228–248. 10.1016/j.trb.2019.05.005

[120] Donovan B, Work DB. Empirically quantifying city-scale transportation system resilience to extreme events. Transp Res Part C Emerg Technol. 2017;79: 333–346. 10.1016/j.trc.2017.03.002

[121] Zhu Y, Ozbay K, Xie K, Yang H. Using big data to study resilience of taxi and subway trips for hurricanes Sandy and Irene. Transp Res Rec. 2016;2599: 70–80. 10.3141/2599-09

[122] Nateghi R. Multi-dimensional infrastructure resilience modeling: An application to hurricane-prone electric power distribution systems. IEEE Access. 2018;6: 13478–13489. 10.1109/ACCESS.2018.2792680

[123] Reed DA, Friedland CJ, Wang S, Massarra CC. Multi-hazard system-level logit fragility functions. Eng Struct. 2016;122: 14–23. 10.1016/j.engstruct.2016.05.006

[124] Konila Sriram LM, Ulak MB, Ozguven EE, Arghandeh R. Multi-network vulnerability causal model for infrastructure co-resilience. IEEE Access. 2019;7: 35344–35358. 10.1109/ACCESS.2019.2904457

[125] Ryu J, Park H. Resilience assessment for interdependent water supply systems based on a system dynamics model. WIT Trans Ecol Environ. 2018;215: 241–245. 10.2495/EID180221

[126] Takagi H, Li S, de Leon M, Esteban M, Mikami T, Matsumaru R, et al. Storm surge and evacuation in urban areas during the peak of a storm. Coast Eng. 2016;108: 1–9. 10.1016/j.coastaleng.2015.11.002

[127] Zarei M, Ashkezari S, Yari M. The investigation of the function of the central courtyard in moderating the harsh environmental conditions of a hot and dry climate (Case study: City of Yazd, Iran). Spatium. 2017;38: 1–9. 10.2298/SPAT1738001Z

[128] Glas H, Deruyter G, De Maeyer P, Mandal A, James-Williamson S. Analyzing the sensitivity of a flood risk assessment model towards its input data. Nat Hazards Earth Syst Sci. 2016;16: 2529–2542. 10.5194/nhess-16-2529-2016

[129] Ali FMM, Ingirige B, Zainal Abidin NA. Assembling and (re)assembling critical infrastructure resilience in Khulna City, Bangladesh. Procedia Engineering. 2018;212: 832–839. 10.1016/j.proeng.2018.01.107

[130] Lin N, Kopp RE, Horton BP, Donnelly JP. Hurricane Sandy’s flood frequency increasing from year 1800 to 2100. Proc Natl Acad Sci USA. 2016;113: 12071–12075. 10.1073/pnas.1604386113 27790992 PMC5087008

[131] Ullman DS, Ginis I, Huang W, Nowakowski C, Chen X, Stempel P. Assessing the multiple impacts of extreme hurricanes in southern New England, USA. Geosciences. 2019;9: 265. 10.3390/geosciences9060265

[132] Suh S-W, Kim H-J. Simulation of wave overtopping and inundation over a dike caused by Typhoon Chaba at Marine City, Busan, Korea. J Coast Res. 2018;85: 711–715. 10.2112/SI85-143.1

[133] Guimarães PV, Farina L, Toldo EE Jr. Analysis of extreme wave events on the southern coast of Brazil. Nat Hazards Earth Syst Sci. 2014;14: 3195–3205. 10.5194/nhess-14-3195-2014

[134] Zhong H, van Overloop P-J, van Gelder PHAJM. A joint probability approach using a 1-D hydrodynamic model for estimating high water level frequencies in the Lower Rhine Delta. Nat Hazards Earth Syst Sci. 2013;13: 1841–1852. 10.5194/nhess-13-1841-2013

[135] Ke Q, Jonkman S, van Gelder P, Bricker J. Frequency analysis of storm-surge-induced flooding for the Huangpu River in Shanghai, China. J Mar Sci Eng. 2018;6: 70. 10.3390/jmse6020070

[136] Orton P, Georgas N, Blumberg A, Pullen J. Detailed modeling of recent severe storm tides in estuaries of the New York City region: storm tides in New York City Estuaries. J Geophys Res. 2012;117: C09030. 10.1029/2012JC008220

[137] Orton P, Talke S, Jay D, Yin L, Blumberg A, Georgas N, et al. Channel shallowing as mitigation of coastal flooding. J Mar Sci Eng. 2015;3: 654–673. 10.3390/jmse3030654

[138] Mercer A, Dyer J. A new scheme for daily peak wind gust prediction using machine learning. Procedia Comput Sci. 2014;36: 593–598. 10.1016/j.procs.2014.09.059

[139] Loftis JD, Forrest D, Katragadda S, Spencer K, Organski T, Nguyen C, et al. StormSense: A new integrated network of IoT water level sensors in the smart cities of Hampton Roads, VA. Mar Technol Soc J. 2018;52: 56–67. 10.4031/MTSJ.52.2.7 31092957 PMC6512834

[140] Kh. Zamim S, Saad Faraj N, A. Aidan I, M. S. Al-Zwainy F, A. AbdulQader M, A. Mohammed I. Prediction of dust storms in construction projects using intelligent artificial neural network technology. PEN. 2019;7: 1659. 10.21533/pen.v7i4.857

[141] Hoffmann P, Merker C, Lengfeld K, Ament F. The Hamburg Tornado (7 June 2016) from the perspective of low-cost high-resolution radar data and weather forecast model. Atmos Res. 2018;211: 1–11. 10.1016/j.atmosres.2018.04.009

[142] Chandrasekar V, Chen H, Philips B. Principles of high-resolution radar network for hazard mitigation and disaster management in an urban environment. J Meteorol Soc Japan. 2018;96A: 119–139. 10.2151/jmsj.2018-015

[143] Carrasco S, Ochiai C, Okazaki K. A study on housing modifications in resettlement sites in Cagayan de Oro, Philippines. J Asian Archit Build Eng. 2016;15: 25–32. 10.3130/jaabe.15.25

[144] Kammerbauer Mark. Asymmetrical recovery in cities after disaster: The lower Ninth Ward in New Orleans after Hurricane Katrina. Raumforsch Raumordn. 2014;72: 427–439. 10.1007/s13147-014-0309-4

[145] Hernández D, Chang D, Hutchinson C, Hill E, Almonte A, Burns R, et al. Public housing on the periphery: Vulnerable residents and depleted resilience reserves post-Hurricane Sandy. J Urban Health. 2018;95: 703–715. 10.1007/s11524-018-0280-4 30088128 PMC6181816

[146] Carrasco S, Ochiai C, Okazaki K. Disaster induced resettlement: Multi-stakeholder interactions and decision making following tropical storm Washi in Cagayan de Oro, Philippines. Procedia Soc Behav Sci. 2016;218: 35–49. 10.1016/j.sbspro.2016.04.008

[147] Gamboa FB. Flooded, resettlements and forgotten: Disaster risk transfer in Motozintla, Chiapas. Revista de Ingeniería. 2010;31: 132–144.

[148] McConnell C, Bertolin C. Quantifying environmental impacts of temporary housing at the urban scale: Intersection of vulnerability and post-hurricane relief in New Orleans. Int J Disaster Risk Sci. 2019;10: 478–492. 10.1007/s13753-019-00244-y

[149] White SS. Out of the rubble and towards a sustainable future: The “greening” of Greensburg, Kansas. Sustainability. 2010;2: 2302–2319. 10.3390/su2072302

[150] Dominoni A, Quaquaro B, Fairburn S. Space4Inspiration: Survival Lab. Designing countermeasures for natural disasters. Des J. 2017;20: S1927–S1937. 10.1080/14606925.2017.1352710

[151] Šakić Trogrlić R, Rijke J, Dolman N, Zevenbergen C. Rebuild by design in Hoboken: A design competition as a means for achieving flood resilience of urban areas through the implementation of green infrastructure. Water. 2018;10: 553. 10.3390/w10050553

[152] Lochhead H. Resilience by design: Can innovative processes deliver more? Procedia Eng. 2017;180: 7–15. 10.1016/j.proeng.2017.04.160

[153] Hoeferlin DJ. The Franz Building: A strong advocation for adaptive re-use in post-Katrina New Orleans. J Green Build. 2009;4: 23–40. 10.3992/jgb.4.1.23

[154] Rohland E. Adapting to hurricanes. A historical perspective on New Orleans from its foundation to Hurricane Katrina, 1718–2005: Adapting to hurricanes. WIREs Clim Change. 2018;9: e488. 10.1002/wcc.488

[155] Scoppetta Cecilia. “Natural” disasters as (neo-liberal) opportunity? Discussing post-hurricane Katrina urban regeneration in New Orleans. Journal of Land Use, Mobility and Environment. 2016;9: 25–41. 10.6092/1970-9870/3725

[156] Kates RW, Colten CE, Laska S, Leatherman SP. Reconstruction of New Orleans after Hurricane Katrina: A research perspective. PNAS. 2006;103: 14653–14660. 10.1073/pnas.0605726103 17003119 PMC1595407

[157] Baade RA, Matheson VA. Professional sports, Hurricane Katrina, and the economic redevelopment of New Orleans. Contemp Econ Policy. 2007;25: 591–603. 10.1111/j.1465-7287.2007.00075.x

[158] Xiao Y, Wu K, Finn D, Chandrasekhar D. Community businesses as social units in post-disaster recovery. J Plan Educ Res. 2018; 1–14. 10.1177/0739456X18804328

[159] Cambaza E, Mongo E, Anapakala E, Nhambire R, Singo J, Machava E. Outbreak of cholera due to Cyclone Kenneth in northern Mozambique, 2019. Int J Environ Res Public Health. 2019;16: 2925. 10.3390/ijerph16162925 31443180 PMC6720437

[160] Raza T C. Rentoy F, Ahmed N, Thess Khas S. Raza AVLA, E. Marasigan KM, M. Espinosa RI. Water challenges and urban sustainable development in changing climate: economic growth agenda for global south. Eur J Sustain Dev. 2019;8: 421. 10.14207/ejsd.2019.v8n4p421

[161] Sim T, Wang D, Han Z. Assessing the disaster resilience of megacities: The case of Hong Kong. Sustainability. 2018;10: 1137. 10.3390/su10041137

[162] Aguilar-Barajas I, Sisto NP, Ramirez AI, Magaña-Rueda V. Building urban resilience and knowledge co-production in the face of weather hazards: Flash floods in the Monterrey Metropolitan Area (Mexico). Environ Sci Policy. 2019;99: 37–47. 10.1016/j.envsci.2019.05.021

[163] Vega JL, Díaz D. Developing sustainable planning for heritage conservation in the tropics: A GIS-based risk and vulnerability assessment profile for historic archives in Puerto Rico. WIT Trans Ecol Environ. 2018;217: 613–623. 10.2495/SDP180521

[164] Safruddim Imran AM, Busthan Pachri. Flood and landslide vulnerability as natural hazard in Parepare City. IOP Conf Ser: Earth Environ Sci. 2019;235: 012079. 10.1088/1755-1315/235/1/012079

[165] Hays WW. Hazard and Risk Assessments in the United States. Episodes. 1991;14: 7–12. 10.18814/epiiugs/1991/v14i1/003

[166] Santos-Burgoa C, Sandberg J, Suárez E, Goldman-Hawes A, Zeger S, Garcia-Meza A, et al. Differential and persistent risk of excess mortality from Hurricane Maria in Puerto Rico: A time-series analysis. Lancet Planet Health. 2018;2: e478–e488. 10.1016/S2542-5196(18)30209-2 30318387

[167] Howland RE, Li W, Madsen AM, Wong H, Das T, Betancourt FM, et al. Evaluating the use of an electronic death registration system for mortality surveillance during and after Hurricane Sandy: New York City, 2012. Am J Public Health. 2015;105: e55–e62. 10.2105/AJPH.2015.302784 26378834 PMC4605157

[168] Eisenman DP, Cordasco KM, Asch S, Golden JF, Glik D. Disaster planning and risk communication with vulnerable communities: Lessons from Hurricane Katrina. Am J Public Health. 2007;97: S109–S115. 10.2105/AJPH.2005.084335 17413069 PMC1855003

[169] Solecki W, Leichenko R, Eisenhauer D. Extreme climate events, household decision-making and transitions in the immediate aftermath of Hurricane Sandy. Misc Geogr. 2017;21: 139–150. 10.1515/mgrsd-2017-0029

[170] Huang X, Wang C, Lu J. Understanding the spatiotemporal development of human settlement in hurricane-prone areas on the US Atlantic and Gulf coasts using nighttime remote sensing. Nat Hazards Earth Syst Sci. 2019;19: 2141–2155. 10.5194/nhess-19-2141-2019

[171] Wang Q, Taylor JE. Patterns and limitations of urban human mobility resilience under the influence of multiple types of natural disaster. Braunstein LA, editor. PLoS ONE. 2016;11: e0147299. 10.1371/journal.pone.0147299 26820404 PMC4731215

[172] Snyder B, Napawan NC. Porosity: Networking cities for a changing climate. Architecture_MPS. 2014;6: 1–18. 10.14324/111.444.amps.2014v6i1.001

[173] Wang Q, Taylor JE. Quantifying human mobility perturbation and resilience in Hurricane Sandy. Wu Y, editor. PLoS ONE. 2014;9: e112608. 10.1371/journal.pone.0112608 25409009 PMC4237337

[174] Wang Q, Taylor JE. Resilience of human mobility under the influence of typhoons. Procedia Eng. 2015;118: 942–949. 10.1016/j.proeng.2015.08.535

[175] Kryvasheyeu Y, Chen H, Obradovich N, Moro E, Van Hentenryck P, Fowler J, et al. Rapid assessment of disaster damage using social media activity. Sci Adv. 2016;2: e1500779. 10.1126/sciadv.1500779 27034978 PMC4803483

[176] Fussell E. The long-term recovery of New Orleans’ population after Hurricane Katrina. Am Behav Sci. 2015;59: 1231–1245. 10.1177/0002764215591181 26880853 PMC4752119

[177] Meduri Y. Multi-stakeholder participation in disaster recovery: A case study. Procedia Eng. 2016;159: 179–185. 10.1016/j.proeng.2016.08.153

[178] Yabe T, Ukkusuri SV, C. Rao PS. Mobile phone data reveals the importance of pre-disaster inter-city social ties for recovery after Hurricane Maria. Appl Netw Sci. 2019;4: 98. 10.1007/s41109-019-0221-5

[179] Dunlap E, Graves J, Benoit E. Stages of drug market change during disaster: Hurricane Katrina and reformulation of the New Orleans drug market. Int J Drug Policy. 2012;23: 473–480. 10.1016/j.drugpo.2012.04.003 22728093 PMC3459295

[180] Gamble JL, Hurley BJ, Schultz PA, Jaglom WS, Krishnan N, Harris M. Climate change and older Americans: State of the science. Environ Health Perspect. 2013;121: 15–22. 10.1289/ehp.1205223 23033457 PMC3553435

[181] Dominianni C, Ahmed M, Johnson S, Blum M, Ito K, Lane K. Power outage preparedness and concern among vulnerable New York City residents. J Urban Health. 2018;95: 716–726. 10.1007/s11524-018-0296-9 30051238 PMC6181821

[182] Bukvic A, Gohlke J, Borate A, Suggs J. Aging in flood-prone coastal areas: Discerning the health and well-being risk for older residents. Int J Environ Res Public Health. 2018;15: 2900. 10.3390/ijerph15122900 30567352 PMC6313428

[183] Gonzalez A, Rasul R, Molina L, Schneider S, Bevilacqua K, Bromet EJ, et al. Differential effect of Hurricane Sandy exposure on PTSD symptom severity: comparison of community members and responders. Occup Environ Med. 2019;76: 881–887. 10.1136/oemed-2019-105957 31615861

[184] Schneider S, Rasul R, Liu B, Corry D, Lieberman-Cribbin W, Watson A, et al. Examining posttraumatic growth and mental health difficulties in the aftermath of Hurricane Sandy. Psychol Trauma. 2019;11: 127–136. 10.1037/tra0000400 30113188 PMC6345600

[185] for the Louisiana Healthy Aging Study, Stanko KE, Cherry KE, Ryker KS, Mughal F, Marks LD, et al. Looking for the silver lining: Benefit finding after Hurricanes Katrina and Rita in middle-aged, older, and oldest-old Adults. Curr Psychol. 2015;34: 564–575. 10.1007/s12144-015-9366-2 27440961 PMC4948298

[186] Sirey JA, Berman J, Halkett A, Giunta N, Kerrigan J, Raeifar E, et al. Storm impact and depression among older adults living in Hurricane Sandy–affected areas. Disaster Med Public Health Prep. 2017;11: 97–109. 10.1017/dmp.2016.189 27995840

[187] West JS, Price M, Gros KS, Ruggiero KJ. Community support as a moderator of postdisaster mental health symptoms in urban and nonurban communities. Disaster Med Public Health Prep. 2013;7: 443–451. 10.1017/dmp.2013.74 24274123 PMC3955699

[188] Gruebner O, Lowe SR, Tracy M, Joshi S, Cerdá M, Norris FH, et al. Mapping concentrations of posttraumatic stress and depression trajectories following Hurricane Ike. Sci Rep. 2016;6: 32242. 10.1038/srep32242 27558011 PMC4997353

[189] Lee DC, Smith SW, Carr BG, Doran KM, Portelli I, Grudzen CR, et al. Geographic distribution of disaster-specific emergency department use after Hurricane Sandy in New York City. Disaster Med Public Health Prep. 2016;10: 351–361. 10.1017/dmp.2015.190 26857616 PMC7112993

[190] Gruebner O, Lowe SR, Sampson L, Galea S. The geography of post-disaster mental health: spatial patterning of psychological vulnerability and resilience factors in New York City after Hurricane Sandy. Int J Health Geogr. 2015;14: 16. 10.1186/s12942-015-0008-6 25889102 PMC4461997

[191] Lowe SR, Sampson L, Gruebner O, Galea S. Psychological resilience after Hurricane Sandy: The influence of individual- and community-level factors on mental health after a large-scale natural disaster. Chao L, editor. PLoS ONE. 2015;10: e0125761. 10.1371/journal.pone.0125761 25962178 PMC4427458

[192] Cepeda A, Saint Onge JM, Kaplan C, Valdez A. The association between disaster-related experiences and mental health outcomes among drug using African American Hurricane Katrina evacuees. Community Ment Health J. 2010;46: 612–620. 10.1007/s10597-009-9286-4 20091228 PMC2919598

[193] Bonanno GA, Galea S, Bucciarelli A, Vlahov D. What predicts psychological resilience after disaster? The role of demographics, resources, and life stress. J Consult Clin Psychol. 2007;75: 671–682. 10.1037/0022-006X.75.5.671 17907849

[194] Thornton E, Kennedy S, Hayes-Watson C, Krouse RZ, Mitchell H, Cohn RD, et al. Adapting and implementing an evidence-based asthma counseling intervention for resource-poor populations. J Asthma. 2016;53: 825–834. 10.3109/02770903.2016.1155219 27049234 PMC5040354

[195] Chulada PC, Kennedy S, Mvula MM, Jaffee K, Wildfire J, Thornton E, et al. The head-off environmental asthma in Louisiana (HEAL) study—methods and study population. Environ Health Perspect. 2012;120: 1592–1599. 10.1289/ehp.1104239 22895349 PMC3556602

[196] Toner ES, McGinty M, Schoch-Spana M, Rose DA, Watson M, Echols E, et al. A community checklist for health sector resilience informed by Hurricane Sandy. Health Secur. 2017;15: 53–69. 10.1089/hs.2016.0079 28192055 PMC5551499

[197] Cohen GH, Tamrakar S, Lowe S, Sampson L, Ettman C, Linas B, et al. Comparison of simulated treatment and cost-effectiveness of a stepped care case-finding intervention vs usual care for posttraumatic stress disorder after a natural disaster. JAMA Psychiatry. 2017;74: 1251–1258. 10.1001/jamapsychiatry.2017.3037 28979968 PMC6583387

[198] Broz D, Levin EC, Mucha AP, Pelzel D, Wong W, Persky VW, et al. Lessons learned from Chicago’s emergency response to mass evacuations caused by Hurricane Katrina. Am J Public Health. 2009;99: 1496–1504. 10.2105/AJPH.2007.126680 19197088 PMC2707481

[199] Chen W, Zhai G, Ren C, Shi Y, Zhang J. Urban resources selection and allocation for emergency shelters: In a multi-hazard environment. Int J Environ Res Public Health. 2018;15: 1261. 10.3390/ijerph15061261 29903997 PMC6025408

[200] Chan E, Man A, Lam H, Chan G, Hall B, Hung K. Is urban household emergency preparedness associated with short-term impact reduction after a super typhoon in subtropical city? Int J Environ Res Public Health. 2019;16: 596. 10.3390/ijerph16040596 30791356 PMC6406516

[201] Hongbo W, Bárcena A, Kituyi M, Akhtar S, Lopes C, Khalaf R, et al. World Economic Situation and Prospects 2016. New York: United Nations; 2016 pp. 1–231.

[202] Shibayama T. 2005 Storm Surge by Hurricane Katrina. In: Esteban M, Takagi H, Shibayama T, editors. Handbook of Coastal Disaster Mitigation for Engineers and Planners. Boston: Butterworth-Heinemann; 2015. pp. 21–34. 10.1016/B978-0-12-801060-0.00002–2

[203] Nel JL, Le Maitre DC, Nel DC, Reyers B, Archibald S, van Wilgen BW, et al. Natural hazards in a changing world: A case for ecosystem-based management. Magar V, editor. PLoS ONE. 2014;9: e95942. 10.1371/journal.pone.0095942 24806527 PMC4012988

[204] Du S, Scussolini P, Ward PJ, Zhang M, Wen J, Wang L, et al. Hard or soft flood adaptation? Advantages of a hybrid strategy for Shanghai. Glob Environ Change. 2020;61: 102037. 10.1016/j.gloenvcha.2020.102037

[205] Watts N, Adger WN, Agnolucci P, Blackstock J, Byass P, Cai W, et al. Health and climate change: Policy responses to protect public health. Lancet. 2015;386: 1861–1914. 10.1016/S0140-6736(15)60854-6 26111439

[206] Watts N, Adger WN, Ayeb-Karlsson S, Bai Y, Byass P, Campbell-Lendrum D, et al. The Lancet Countdown: Tracking progress on health and climate change. Lancet. 2017;389: 1151–1164. 10.1016/S0140-6736(16)32124-9 27856085

