# Supplementary material for: Wind disasters adaptation in cities in a changing climate: A systematic review
Source: PLoS One. 2021 Mar 17;16(3):e0248503. doi: 10.1371/journal.pone.0248503 (PMC7968717; doi:10.1371/journal.pone.0248503)
Supplement: S1 Appendix — (DOCX) [file pone.0248503.s005.docx]

| **City Synonyms** | **Wind Hazard Synonyms** | **Adaptation Synonyms** |
| --- | --- | --- |
| “urban” OR “city” OR “cities” OR “municipal” | “wind AND hazard*” OR “wind AND risk*” OR ”wind AND damage*” OR “wind AND disaster* NOT snow” OR “hurricane$” OR “typhoon$” OR “cyclone$” OR “tropical cyclone$” OR “tropical depression” OR “tropical storm$” OR “extratropical cyclone$” OR “tornado*” OR “severe wind$” OR “sandstorm$” OR “dust storm$” | “adapt*” OR “vulnerab*” OR “resilien*” OR “fragil*” |
| **Dataset** | **Search Query** | |
| Web of Science | TS=adapt* **OR** TS=("vulnerab*" OR "resilien*" OR "fragil*") **AND** TS=("urban" OR "city" OR "cities" OR "municipal*" ) **AND** TS=((wind AND hazard*) OR (wind AND risk*) OR (wind AND damage*) OR (wind AND disaster*) NOT snow) **OR** TS=("hurricane$" OR "typhoon$" OR "cyclone$" OR "tropical cyclone$" OR "tropical depression" OR "tropical storm$" OR "extratropical cyclone$" OR "tornado*" OR "severe wind$" OR "sandstorm$" OR "dust storm$") | |
|  |  | |
| Scopus | TITLE-ABS-KEY (adapt*) **OR** TITLE-ABS-KEY (vulnerab*) OR TITLE-ABS-KEY (resilien*) OR TITLE-ABS-KEY (fragil*) **AND** TITLE-ABS-KEY (urban) OR TITLE-ABS-KEY (city) OR TITLE-ABS-KEY (cities) OR TITLE-ABS-KEY ( municipal*) AND (LIMIT-TO (ACCESSTYPE(OA))) **AND** TITLE-ABS-KEY (wind AND (hazard OR risk OR damage OR disaster) AND NOT snow) AND (LIMIT-TO (ACCESSTYPE(OA))) **OR** TITLE-ABS-KEY (hurricane OR typhoon OR cyclone OR "TROPICAL CYCLONE" OR "TROPICAL DEPRESSION" OR "TROPICAL STORM" OR "EXTRATROPICAL CYCLONE" OR tornado OR "SEVERE WIND" OR sandstorm OR "DUST STORM") AND (LIMIT-TO (ACCESSTYPE(OA))) | |

**Example for explaining the topic modeling**

In a process of extracting three topics, the output for one article is 33% of topic 1, 45% of topic 2, and 21% of topic 3. Topics are formed by words that can be organized to make them tell meaningful things. A group of words “resilience”, “urban”, and “system” can be understood as “the resilience of urban systems”. Further conclusion is that this article is most likely discussing about topic 2, with some fewer contents relating to topic 1 and topic 3.
