# Supplementary material for: Wind disasters adaptation in cities in a changing climate: A systematic review
Source: PLoS One. 2021 Mar 17;16(3):e0248503. doi: 10.1371/journal.pone.0248503 (PMC7968717; doi:10.1371/journal.pone.0248503)
Supplement: S2 Appendix — (DOCX) [file pone.0248503.s006.docx]

| **Regions** | **Coastal** | **Cities** | **New York** | **New Orleans** | **Hurricane Sandy** | **Case study** | **General discussion** | **Academic articles** |
| --- | --- | --- | --- | --- | --- | --- | --- | --- |
| Which regions are studied by the paper? | Whether the studied area belongs to coastal regions. | Which cities are mentioned in the paper? | Is New York City one of the cities being studied? | Is New Orleans one of the cities being studied? | Is hurricane Sandy one of the major focuses of wind hazards in the paper? | Is this paper focused on case studies? | Does this paper talk about relevant topics generally? | Is this paper an academic peer-reviewed article? |

| **Engineered and built environment** | **Technological** | **Ecosystem** | **Services** | **Educational** | **Informational** | **Behavioral** | **Economic** | **Laws and regulations** | **Government policies and programs** |
| --- | --- | --- | --- | --- | --- | --- | --- | --- | --- |
| Referring to the IPCC fifth report, insert ‘🗸‘ in the column if there is a corresponding adaptation measure mentioned in the paper. | | | | | | | | | |
